# Sliding window based rare partial periodic pattern mining algorithms over temporal data streams

**DOI:** 10.3389/fdata.2025.1600267

**Published:** 2025-06-04

**Authors:** K. Jyothi Upadhya, Ronan Lobo, Mini Shail Chhabra, Aman Paleja, B. Dinesh Rao, Geetha M., Prachi Sisodia, Bolusani Akshita Reddy

**Affiliations:** ^1^Department of Computer Science and Engineering, Manipal Institute of Technology, Manipal Academy of Higher Education, Manipal, Karnataka, India; ^2^Manipal School of Information Sciences, Manipal Academy of Higher Education, Manipal, Karnataka, India; ^3^Department of Electronics and Communication, Manipal Institute of Technology, Manipal Academy of Higher Education, Manipal, Karnataka, India

**Keywords:** partial periodic mining, rare partial periodic pattern mining, rare periodic pattern mining, stream periodic pattern mining, tree-based stream mining, list-based stream mining

## Abstract

Periodic pattern mining, a branch of data mining, is expanding to provide insight into the occurrence behavior of large volumes of data. Recently, a variety of industries, including fraud detection, telecommunications, retail marketing, research, and medical have found applications for rare association rule mining, which uncovers unusual or unexpected combinations. A limited amount of literature demonstrated how periodicity is essential in mining low-support rare patterns. In addition, attention must be placed on temporal datasets that analyze crucial information about the timing of pattern occurrences and stream datasets to manage high-speed streaming data. Several algorithms have been developed that effectively track the cyclic behavior of patterns and identify the patterns that display complete or partial periodic behavior in temporal datasets. Numerous frameworks have been created to examine the periodic behavior of streaming data. Nevertheless, such a method that focuses on the temporal information in the data stream and extracts rare partial periodic patterns has yet to be proposed. With a focus on identifying rare partial periodic patterns from temporal data streams, this paper proposes two novel sliding window-based single scan approaches called *R3PStreamSW-Growth* and *R3PStreamSW-BitVectorMiner*. The findings showed that when a dense dataset *Accidents* is considered, for different threshold variations *R3P-StreamSWBitVectorMiner* outperformed *R3PStreamSW-Growth* by about 93%. Similarly, when the sparse dataset *T10I4D100K* is taken into account, *R3P-StreamSWBitVectorMiner* exhibits a 90% boost in performance. This demonstrates that on a range of synthetic, real-world, sparse, and dense datasets for different thresholds, *R3P-StreamSWBitVectorMiner* is significantly faster than *R3PStreamSW-Growth*.

## 1 Introduction

In this data-centric era, various applications use frequent pattern mining (FPM). This data mining technique discovers frequently co-occurring patterns based on user-specified thresholds in static as well as stream data. Streaming data are unbounded in quantity and change very fast. Sensor data, Stock tickers, Telecommunication call data, Credit card transactions and Internet packet streams are all considered online data that have to be processed as fast as possible because of their rapid arrival. The swift rise of these novel application fields has made it more challenging to perform sophisticated analysis and data mining across massive, rapidly emerging data streams to identify engrossing trends, patterns, and outliers. Compared to static mining algorithms, the models designed to handle the stream data have to consider the following essential concerns: (i) The stream data have to be scanned once and need not be backtracked. (ii) Stream data have to be processed as fast as possible by considering memory limitations. (iii) Because of dynamic behavior, frequent patterns may become gradually infrequent and vice versa. Considering these issues, several frequent stream mining algorithms have been designed to successfully extract frequent patterns from streaming data. Data stream processing models are divided into Landmark, Sliding and Damped window models. An excerpt of the data stream is called a window (Cheng et al., 2008; Borah and Nath, 2017a). The landmark window model performs data extraction by considering the data within the landmark and the current time and maintaining summary data (Manku and Motwani, 2002; Lee and Lee, 2005). The sliding window model focuses on the current window part of the stream data. It processes new transactions by sliding the window and deletes the older transactions (Tanbeer et al., 2010, 2017; Li and Lee, 2009; Tanbeer et al., 2009; Lee et al., 2014). The time fading or damped window model considers the order of appearance of data and depending on this, allocates various weights to the data. Older transactions will have less weightage, whereas the recent data will have higher weights (Tsai, 2009; Hung et al., 2015). Applications that manage stream-oriented data typically value research focusing on recent data. In this direction, the sliding window model is a technique that restricts the stream data to a current window.

On the other side, Rare Pattern Mining (RPM) successfully uncovers the hidden, uncommon or unexpected behavior that FPM fails to extract. When the medicine field is considered, rare responses which may show adverse reactions are sometimes more interesting than the expected, common responses to medications (Koh and Ravana, 2016). Further, several algorithms (Huang et al., 2015, 2014; Koh and Ravana, 2016) are designed which are capable of mining rare patterns from stream data as well. However, these pattern mining techniques have concentrated on the support metrics.

Periodic Frequent Pattern Mining (PFPM) then arose as a significant area that demonstrated the value of considering occurrence behavior also known as periodicity or regularity into account during the pattern mining process. PFPM found its usage in an extensive range of applications, such as the study of gene and medical data (khaleel et al., 2015; Glynn, 2006), online user behavior (Yi et al., 2018), mobility intention (Fong et al., 2011), and so forth. The algorithms designed in this area are able to extract periodic frequent patterns from static datasets. Additionally, several algorithms (Tanbeer et al., 2010, 2017; Mesama and Amphawan, 2018; Rashid et al., 2013) have been designed to focus on the occurrence behavior of frequently occurring patterns in the stream data.

Initial PFPM models have shown strict behavior on the cyclic repetitions of the patterns by eliminating those patterns even when a single periodicity does not confirm the threshold measure under consideration. However, real-life applications show a requirement for relaxation in the strictness measure during the extraction of periodic patterns. For example, heavy traffic is observed on weekends rather than weekdays. In the retail market, “bread” and “butter” is purchased regularly. Meanwhile, “jam” and “rice” may be purchased monthly. Partial Periodic Pattern Mining (PPPM), relaxed the strictness measure of periodicity by permitting users to choose the minimal cyclic repetitions allowed when extracting a partial periodic pattern. In addition, recent research (Upadhya et al., 2023; Kiran et al., 2017b, 2022b; Ravikumar et al., 2022, 2021) has also shown the need of taking temporal information into account. For instance, compared to other timings, traffic congestion can be worse between 9 a.m. to 10 a.m. and 5 p.m. to 6 p.m. Temporal datasets possess characteristics such as: (i) The timestamp information from the incoming database is used to sort temporal datasets. (ii) There is a possibility for inconsistent timing between subsequent transactions. (iii) Several transactions may arrive simultaneously. Additionally, merging these transactions could result in the loss of the actual support data and the creation of misleading associations (Upadhya et al., 2023; Kiran et al., 2022b).

“Periodic Rare Pattern Mining” (PRPM) has been growing as a promising area with a focus on discovering unusual or unexpected combinations that are overlooked by PFPM algorithms. Sometimes, rare patterns occur in the entire transaction dataset, they may be periodic and significant. Few models have been developed to extract these patterns (Fournier-Viger et al., 2020; Jyothi et al., 2023). However, dealing with the timestamp data will enhance the knowledge extracted. For example, Kiran et al. (2022b) considered a case study of the raw traffic congestion data by combining the road segments that had congestion above 300 meters, which were gathered by sensors connected to Japan's spatial locations. It produced a collection of highly crowded road segments where people frequently encountered traffic jams. Conversely, at certain times, heavy traffic may be observed during festival days. Festivals occur throughout the year despite their sporadic nature. Accordingly, it is important to extract the timestamp information. In contrast to periodic frequent patterns, these patterns have low support counts and greater periodicities. This information on highly congested roads could assist traffic control rooms in directing traffic, recommending police patrols, and warning pedestrians on the roads for those who frequently encounter traffic congestion on festival days. In addition, to forecast on-road congestion segments, several statistical and machine learning models (Nguyen et al., 2019) have been designed. The proposed model could be used to analyze the data generated by these prediction models to identify sets of extremely congested road segments during festival days. Most PFPM algorithms fail to record the occurrence of these patterns. With this motivation, the following major contributions are proposed in this paper:

- 3P-Growth is a novel model designed by Kiran et al. (2022b, 2017a), where partial periodic patterns are extracted from temporal static data using a pattern-growth approach. Here 3P-Tree plays a major role in capturing temporal information. The tree is created by scanning the database twice, which is inappropriate for mining stream data. In this direction, 3P-Growth is enhanced and a novel sliding window-based single scan algorithm named *R3PStreamSW-Growth* is presented, which successfully captures Rare Partial Periodic Patterns (R3Ps) from temporal stream data. The major components of this algorithm are *R3PStream-List, R3PStream-Tree* and *R3PStream-Queue*.
- Concerning the generation of partial periodic one-length patterns from the current timestamp window, a *R3PStream-List* structure is maintained. Subsequently, it is refreshed, which helps in pruning the aperiodic one-length patterns and further reduction of the extensive search space.
- A highly efficient *R3PStreamSW-Tree* is built that successfully gathers timestamped data from the current window stream. Older transactions are deleted when the window slides, and a fresh set of stream data is added to the *R3PStreamSW-Tree*. A Queue structure called *R3PStream-Queue* accelerates window sliding by directing the traversal to the nodes of *R3PStreamSW-Tree* that hold timestamp data.
- During the mining phase, *R3PStreamSW-Growth* employs a divide-and-conquer strategy, which generates a massive number of conditional pattern trees. This recursive process affects the mining performance. To overcome this, our previous work R3P-BitVectorMiner (Upadhya et al., 2023) is enhanced and a novel depth-first search framework named *R3P-StreamSWBitVectorMiner* is proposed to extract entire R3Ps from the temporal stream data. The current window stream data are transformed into bit-vector and stored in an efficient data structure named R3PStreamSWTSList which helps in pruning non-periodic itemsets.
- Periodic (or cyclic) refers to a temporal stream pattern that satisfies *maxPer*, a user-given periodicity measure. Further, the necessary count of cyclic repetitions is supervised by using two distinct support thresholds, *minFreqPS* and *minRarePS*. In this case, *minRarePS* helps to eliminate the rare patterns that are noisy itemsets that are associated by chance.
- In addition, to maintain the sliding window, window size—*TSWindowSize* and batch size—*TSBatchSize* values are accepted by the user. Further, *mineBSize*, a user-specified value, decides after how many batches of sliding window process the mining happens.
- Several synthetic as well as real-life datasets are considered for experimentation. Additionally, several analyses using wide range of periodicities and support thresholds are presented. Research shows that R3P-StreamSWBitVectorMiner is highly time and space efficient compared to R3PStreamSW-Growth.

The rest of the paper is arranged as follows: Section 2 presents the literature work carried out in the area of PFPM and RPM. Necessary definitions required to enhance the proposed methods are given in Section 3. Section 4 exhibits various modules and discussions of *R3PStreamSW-Growth*. Various modules of *R3P-StreamSWBitVectorMiner* depicted in Section 5. Result analysis and experimental evaluation by considering different datasets are depicted in Section 6. The conclusion and future directions are presented in Section 7.

## 2 Related work

### 2.1 Periodic frequent pattern mining (PFPM)

PFPM focuses on how periodically the patterns occur. Here the literature work is conducted on static and stream data which includes temporal information along with periodicity.

#### 2.1.1 Related work considering periodicity measure in static/stream data

Tanbeer et al. (2008) first presented *Regular Pattern Tree* in which the support information maintained in every node of FP-Tree (Han et al., 2000) was replaced with the transaction id (tid) information in only the leaf node. The tid information aided in the calculation of pattern regularity and was controlled by the threshold *maxPer*. Further, *Regular Pattern Tree* was enhanced to study the regularity of patterns from the data stream (Tanbeer et al., 2010) and body sensor networks (Tanbeer et al., 2017). As these models discarded all the patterns having even one single periodicity larger than *maxPer* threshold several models started replacing *maxPer* with other periodic measures. By using variance as the periodicity measure, Rashid et al. found frequent patterns regularly occurring in the static (Rashid et al., 2012) as well as wireless sensor networks (Rashid et al., 2013). Kiran et al. (2016); Kiran and Kitsuregawa (2014); Venkatesh et al. (2018) handled “rare item problem” by taking unique support as well as periodicity thresholds for each item to extract frequent and rare patterns occurring regularly in a set of transactions. Instead of taking a single periodicity measure, Fournier-Viger et al. (2017) designed *Periodic Frequent Pattern Miner*, which makes use of a combination of minimum, average and maximum regularity thresholds to extract regular frequent itemsets from the static data. *Lability* is a novel measure introduced by Fournier-Viger et al. (2019) which successfully mined periodic patterns that are stable in the database. As setting the appropriate occurrence frequency measure is a difficult task, numerous algorithms found a solution by taking a simple parameter *k* to find regularly present top-k frequent patterns in the database. To mine top-k frequent stable periodic patterns Fournier-Viger et al. (2021) designed a model named *TSPIN*. It built a stable periodic-frequent tree and followed a pattern-growth method to extract the patterns. To mine frequently occurring top-k regular patterns, a single scan algorithm focused on partition and estimation methods was developed by Amphawan et al. (2012). A sliding window technique, *TFRIM-DS* is a contribution of Mesama and Amphawan (2018). This single-scan algorithm successfully mines top-k patterns with highest support and regularly occurring itemsets in the current data stream window. To cope with the huge redundant patterns generated, Amphawan and Lenca (2015) developed a single scan algorithm named *TFRC-Mine* to produce longer non redundant top-k frequent closed itemsets occurring regularly in the dataset. *Closed Regular Patterns in Data Streams* is a contribution of Marriboyina and Reddy (2013) which works in vertical data format using a sliding window model. In the initial phase, it extracts regular patterns and in the next phase, it mines closed regular patterns. Jammalamadaka and Budaraju (2025) considered a medical data stream and identified all negative associations by groping the medications given to patients using a window model. With optimum frequency and regularity, they reduced the number of negative associations from 0.73 to 0.43 when a 1,000 set of items is considered for mining. This helps doctors save patients from the devastating effects. Ishita et al. (2022) designed *RHusp, RIncHusp* and *RStreamHusp* to mine regular high-utility sequential patterns from static, incremental as well as evolving stream data respectively. To uncover regular high-utility itemsets in incremental database systems, Incremental Periodic High-Utility Itemset Miner (IPHM) (Huang et al., 2024) employs a novel incremental utility-list structure. This approach identifies recurrent consumer buying patterns that are typical in everyday situations.

Most of the above methods impose a strict measure on the occurrence behavior and discard a large number of patterns immediately when a single periodicity fails to satisfy the periodicity value considered. To relax this, *PPPM* algorithms are designed which uses a measure to control the minimum count of cyclic repetitions required. A periodic-ratio threshold is used by *GPF-growth*, which is a contribution of Kiran et al. (2017c). This threshold takes care of the proportion of cyclic repetitions of regular itemsets in static databases. Further, to find frequent patterns involving both rare and frequent periodicity, “*Extended Periodic-Frequent pattern-growth”* a pattern-growth approach is designed by Venkatesh et al. (2016) Here the “rare item problem” is solved by utilizing all-confidence as well as periodic-all-confidence measures.

#### 2.1.2 Related work considering temporal information

The algorithms dealing with timestamp information must handle multiple arrival of transactions at a common timestamp and non-uniform occurrence of transactions. *3P-Growth* is a contribution of Kiran et al. (2022b, 2017a) which successfully dealt with temporal datasets. Instead of maintaining tid information, timestamp information is kept track using *3P-list* and *3P-tree* data structures. A pattern growth approach enumerates entire *PPPs* existing throughout the dataset by utilizing the temporal information. A relative periodic-support measure is utilized by Kiran et al. (2017b) to enumerate *PPPs* from the dataset exhibiting non-uniform periodic nature. Further, to extract *PPPs* in non-uniform temporal databases, with a combination of both rare as well as frequent patterns, Kiran et al. (2022a) proposed *G3P-growth*. Here, temporal information is captured in “*G3P-tree”* and this compact tree is recursively mined to extract *PPPs* using relative periodic support measure. To discover periodically correlated patterns that are frequent, Venkatesh et al. (2018) came up with a model named “*Extended Periodic-Correlated pattern-growth”*. As these algorithms dealt with row temporal databases, there are few algorithms designed that consider columnar datasets with timestamp information. To extract frequent patterns that are periodic, a run-time and memory-efficient method named “*Frequent-Equivalence CLass Transformation”* is designed by Ravikumar et al. (2021). An enhancement of *ECLAT* algorithm named *3P-ECLAT* is developed by Ravikumar et al. (2022); Pamalla et al. (2023). At first, *TS-list* is utilized to keep the timestamp information of one length *PPPs*. Next, entire *PPPs* are extracted by performing an intersection operation on the itemsets present in TS-list in a depth-first search manner. To extract maximal *PPPs* and stable periodic frequent patterns from timestamp dataset, Likhita et al. presented *max3P-Growth* (Likitha et al., 2021) and *SPP-ECLAT* (Dao et al., 2023) respectively. Further, setting *minSup* threshold measure is a time consuming task, to overcome it, Likhitha et al. (2023) contributed “*Top-k Periodic-Frequent Pattern Miner”*. The model finds *k* frequent itemsets with lowest periodicities in a dataset with timestamp information. Even though the regularity can be found using PFPM techniques, the study is focused only on frequent patterns. Conversely, the periodicity of rare itemsets is not emphasized in the majority of current PFPM techniques.

### 2.2 Rare itemset mining

#### 2.2.1 Related work in static/stream data without considering periodicity measure

The RPM algorithms can be categorized as Apriori based and Tree based. There are several Apriori based approaches (Adda et al., 2012; Troiano et al., 2009; Troiano and Scibelli, 2014) designed to extract rare itemsets which traverse the itemset lattice in a top-down manner. Initially, longest or k-itemset is constructed. Subsequently, in every level its subsets are found by pruning frequent and noisy itemsets in the dataset. “*NII-Miner”*, is the initial tree-based contribution of Lu et al. (2020) which uses a top-down depth-first strategy to mine uncommon itemsets. The *A*RIMA algorithm, designed by Szathmary et al. (2010) mines all rare itemsets in a static dataset. To deal with the spurious low support threshold patterns, Bouasker and Ben Yahia (2015); Bouasker et al. (2012) designed *CORI* algorithm. In the initial phase, the equivalent vertical bit-wise representation of the input database happens and then all set of correlated rare itemsets are enumerated in a bottom-up method by performing simple logical operations. Minimizing the search space is a major challenge that RPM algorithms have to face. In this regard few algorithms mined sub-range of rare itemsets in a bottom-up strategy by selecting the transactions having minimum one rare item. Tsang et al. (2011) initially built a tree structure named “*Rare Pattern Tree” (RP-Tree)*. Next, a pattern-growth approach is applied to mine the rare itemsets between thresholds *minFrepSup* and *minRareSup*. Based on the concept of *RP-Tree* Huang et al. (2015) constructed *Streaming Rare Pattern Tree* to deal with stream data. A single scan algorithm is built which discovers all the rare itemsets using a sliding window approach with different window and block sizes. Similarly, “Hyper-Linked Rare Pattern Mining” is a novel work of Borah and Nath (2017b). A memory-based queue structure comprising hyper-linked pattern is utilized to mine subset of rare itemsets. To handle both rare as well as frequent itemsets Borah and Nath (2018) and Rai et al. (2022) constructed *Single Scan Pattern Tree* and BIN-Tree respectively. Huang et al. (2014) developed “*Rare Pattern Drift Detector”* which detects drifts in the rare itemsets selected between two threshold values *minFreqSupp* and *minRareSupp*. The associations in a stream data for a particular item during a specific time is tracked by a novel measure called *M*. However, the occurrence behavior is overlooked in these RPM methods.

#### 2.2.2 Related work with periodicity threshold measure

Studying periodic nature of rare itemsets further extracts the significant information in numerous applications. Fournier-Viger et al. (2020) designed *MRCPPS*, a novel framework that extracts rare correlated itemsets that exists throughout various sequences. In combination with support threshold, standard deviation of periods plays a major role in periodicity measure. In addition, the extraction process of periodic rare correlated itemsets also produces a lot of spurious patterns and these patterns are pruned using the bond measure. Our novel work, *PRCPMiner* (Jyothi et al., 2023) is able to enumerate periodic rare itemsets that are correlated in static datatset. Here *CORI* algorithm is modified to deal with periodic behavior. This is achieved by using periodicity threshold in combination with support and bond thresholds. In order to enumerate periodic rare itemsets from the static database, we enhanced *NII-Miner* and proposed *PRPNegTidTreeMiner* algorithm. Here *NegTidTree* is built which serves the dual purpose of finding periodicity information along with support measure. The literature presents very few frameworks have focused on studying the periodic nature of rare itemsets. However, these algorithms have not concentrated on the temporal information. In this regard, *PRCPMiner* is enhanced to design “*3P-BitVectorMiner”* (Upadhya et al., 2023). The model successfully considers timestamp information and examines the occurrence behavior of partial patterns. Further, “*RFPP-BitVectorMiner”* and “*‘R3P-BitVectorMiner”* were presented to extract rare full periodic and partial periodic respectively. Literature shows there is no algorithm designed to extract rare partial periodic patterns from the stream data. With this motivation, to deal with the temporal stream data tree-based and list-based sliding window frameworks are presented in this paper.

## 3 Stream rare partial periodic pattern model

In an application domain, let ψ = {*d*_1_, *d*_2_,…., *d*_*n*_} be a complete collection of items that represent unit of information. Let a temporal transaction *t*_*i*_ = (*Tid, TS, I*) where transaction identifier is represented by a distinct value *Tid*, time stamp is presented by *TS. I* ⊆ ψ is named an itemset (or a pattern). An itemset *I* comprising of *x* unique items, where 1 ≤ *x* ≤ *n* is framed as *x*-pattern. A temporal data stream *TDS* over ψ is an infinite group of ordered transactions i.e., *TDS* = [*t*_1_, *t*_2_,…..,*t*_*p*_). A window *TSW* consists of group of all the transactions between *j*^*th*^ and *k*^*th*^ arrival where 1 ≤ *j* ≤ *k* ≤ *p*. The set of transactions between *j*^*th*^ and *k*^*th*^ arrival forms the size of the window which is defined as *size (TSW)* and is equal to *(k*- *j)*. Let the lower and higher values of time stamp in *TSW* be represented, respectively, by *tsw*_*min*_ and *tsw*_*max*_. The sample temporal stream dataset represented in Table 1 shows that there may be a delay among two consecutive time stamps and two transactions may occur at the same time. Hence, (*tsw*_*max*_ - *tsw*_*min*_ + 1) might not represent |TSW|. If a transaction *t*_*i*_ = (*Tid, TS, I*) occurs in current window *TSW*, the time stamp value of an itemset *P* ∈ *I* can be expressed as *tsw*^*P*^. Let *TSW*^*P*^ be equal to {$tsw_{i}^{P}$,$tsw_{j}^{P}$,…..,$tsw_{q}^{P}$}, where i ≤ *j* ≤ *q* presents the ordered time stamp values in which *P* occurs in current window *TSW*. Support count of *P* is expressed as SupTSW (P)= |*TSW*^*P*^| and represents the group of transactions in which *P* appears in current window *TSW*. Let *TSBatchSize* be the count of transactions slided every time from the current window *TSW*.

**Table 1:** Sample temporal stream dataset—*TDS*.

		**TID**	**Time stamp**	**Items**
I Window		T1	1	m, n, o
		T2	3	q, r, s
	II Window	T3	3	m, n, o, r
		T4	4	m, n, o, r, s
		T5	5	m, p, s
		T6	6	m, n, o, p, r
		T7	7	m, r, s

**Example 1:** Table 1 presents temporal transactions in a data stream *TDS*. Let the window size *TSWindowSize* and batch size *TSBatchSize* be 5 and 2 respectively. The data itemset ψ = {m, n, o, p, q, r, s} comprises 7 unique items. The first transaction *t*_1_ = (*T1, 1, mno*) where *T1* and *1* presents *Tid* and time stamp values respectively and {m, n, o} is a 3-pattern set. In the first time stamp window *tsw*_*min*_ and *tsw*_*max*_ ranges from 1 to 5. Consider the first window, where the itemset {mo} exist in transactions having time stamp values 1, 3 and 4. This leads to *TSW*^*mo*^ = {1,3,4} and SupTSW (mo) = |*TSW*^*mo*^| which results in 3.

**Definition 3.1**. (Periodicity of pattern *P* in current time stamp window ***TSW***) Consider a window TSW, where ($tsw_{i}^{P}$,$tsw_{j}^{P}$) represents a pair of continuous time stamps in *TSW*^*P*^. The time difference among ($tsw_{j}^{P}$ - $tsw_{i}^{P}$) is known as an inter-arrival time, and it is expressed as *iatw*^*P*^. Assuming that the set of inter-arrival times for P in current window TSW is denoted by *IATW*^*P*^ = {$iatw_{1}^{P}$,$iatw_{2}^{P}$,…..,$iatw_{s}^{P}$} where s = (SupTSW (P) - 1). In the current window TSW, when the inter-arrival time of P is no more than the user-specified maximum periodicity threshold i.e. $iatw_{i}^{P}$ ≤ *maxPer*, then it is regarded as periodic in the current window.

**Definition 3.2**. (Periodic support count of pattern *P* in current time stamp window ***TSW***) Let list of all inter-arrival times of an itemset P that are periodic in current window TSW be denoted as $\hat{IATW^{P}}$. Therefore, $\hat{IATW^{P}}$⊆*IATW*^*P*^ such that ∃$iatw_{i}^{P}$∈*IATW*^*P*^ and $iatw_{i}^{P}$ ≤ maxPer, then $iatw_{i}^{P}$∈$\hat{IATW^{P}}$. The periodic support of P in current window TSW denoted as PSTSW (P) and it is equal to |$\hat{IATW^{P}}$|. While selecting the pattern in the current window, the importance is given to both inter-arrival time as well as support count.

**Example 2:** The beginning time stamp values of an itemset {mo} are 1 and 3 resulting in $iatw_{1}^{mo}$ = 2 (3-1) which is considered as its first inter-arrival time. Similarly, the further inter-arrival times are calculated in the first window which results in *IATW*^*mo*^ = {2,1}. If the user entered *maxPer* threshold is assumed as 2, then the $\hat{IATW^{mo}}$ ={1,2} resulting PSTSW (mo) = 2 in the first window.

Here two different support thresholds *minFreqPS* and *minRarePS* thresholds are used along with *maxPer* threshold to control the number of cyclic repetitions. The *minRarePS* threshold assist in discarding the uncommon patterns that are associated by chance and are considered to be noisy itemsets. Based on the strictness of periodicity measure rare periodic patterns can be classified as full and partial periodic patterns.

**Definition 3.3**. (Rare full periodic pattern Q in current time stamp window ***TSW***) Given the user-specified minimum period support thresholds minFreqPS and minRarePS, a pattern Q is said be rare full periodic pattern (RFPP) in current time stamp window TSW, if ((PS (Q) < minFreqPS ∧ PS (Q) ≥ minRarePS) ∧ (PS (Q) = Sup (Q) - 1)).

The Rare full periodic pattern measure is too strict and a pattern is discarded even when one inter-arrival time is also exceeding the *maxPer* threshold. As rare patterns show the tendency to behave non-periodic in certain time-period there is a need to propose a relaxed measure.

**Definition 3.4**. (Rare partial periodic pattern *Q* in current time stamp window ***TSW***) Given the user-specified minimum period support thresholds minFreqPS and minRarePS, a pattern Q is said be Rare Partial Periodic Pattern (R3P) in current time stamp window TSW, if ((PS (Q) < minFreqPS) ∧ (PS (Q) ≥ minRarePS)).

**Problem Definition:** When a temporal data stream *TDS*, a periodicity measure *maxPer* and support measures *minFreqPS* and *minRarePS* are given as input, the process of finding all rare partial periodic itemsets in current time stamp window *TSW* is to output the entire collection of itemsets satisfying the condition specified in Definition 3.4 in current time stamp window *TSW*.

## 4 Rare partial periodic pattern stream mining based on sliding window pattern growth: R3PStreamSW-Growth—A tree-based framework

The tree-based state-of-the-art algorithm 3P-Growth (Kiran et al., 2022b, 2017a) is a two-scan algorithm designed to mine PPPs from temporal static data. However, the requirement of multiple scans is a limitation for mining the stream data. Here, 3P-Growth is enhanced and a new framework called R3PStreamSW-Growth is designed to mine the stream data. The proposed single scan, a pattern-growth method, which is based on a sliding window model, is suitable to discover an entire set of R3Ps over the stream data. This section initially discusses the structure and construction of three components of R3PStreamSW-Growth, namely R3PStreamSW-List, R3PStreamSW-Tree and R3PStreamSW-Queue respectively. Next, the task of inserting the current batch of transactions by removing the old set of transactions is discussed. This process keeps the sliding window always in ready to mine state. Lastly, how the three components collectively aid in mining R3Ps from the stream data is described.

### 4.1 Structure of different components of R3PStreamSW-Growth

#### 4.1.1 Structure of R3PStreamSW-Tree

**Figure 1:**
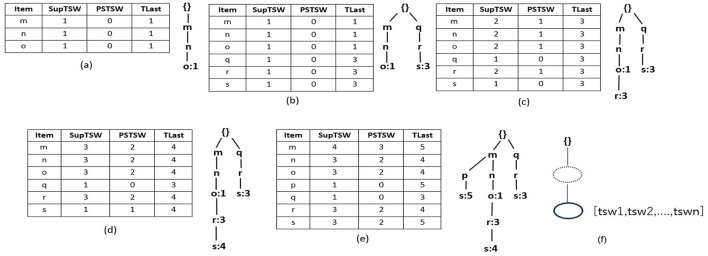
R3PStreamSW-List and R3PStreamSW-Tree. **(a)** After scanning T1. **(b)** After scanning T2. **(c)** After scanning T3. **(d)** After scanning T4. **(e)** After scanning T5-Final R3PStreamSW-List and R3PStreamSW-Tree created for first window of Table 1. **(f)** Structure of R3PStreamSW-Tree.

R3PStreamSW-Tree consists of a “*NULL”* root-node and a group of item-prefix sub-trees, which are stored as branches of the root-node. A unique transaction of current timestamp window *TSW* is presented by every path of the item-prefix sub-tree, and similar to *RPS-Tree* (Tanbeer et al., 2010), the common paths are shared. *ILabel* field of each child node *c* presents a unique item of the current window transaction. The main purpose of R3PStreamSW-Tree is to keep track of the timestamp information. Therefore, instead of storing the transaction-id like RPS-tree, here, the timestamp information is preserved only in the leaf node. The nodes of an R3PStreamSW-tree, except the root node, can be divided into two types, namely tail nodes and ordinary nodes presented by a dotted ellipse and a solid ellipse, respectively, as depicted in Figure 1f. The ordinary nodes maintain two fields *ILabel*, which preserves the item information, and *NodeLink*, which points to the next node in a R3PStreamSW-Tree with matching *ILabel*. The tail nodes maintain an additional field *TSWList* represented in the form [*tsw*_1_,*tsw*_2_,….,*tsw*_*n*_] serve the aim of discovering timestamp information. If transaction *t*_*x*_ = {*i*_1_,*i*_2_,….,*i*_*Tail*_}, then *ILabel* field of tail node represents the item *i*_*Tail*_. Whereas, the *TSWList* field denotes all the timestamp information of the transactions in the current window in which *i*_*Tail*_ is the tail node.

**Lemma 4.1**. A *leaf node* in a R3PStreamSW-Tree inherits the properties of an ordinary node and not vice versa.

**Proof** In a R3PStreamSW-Tree, the item information and *NodeLink* pointers are preserved by an ordinary node. Additionally, it maintains children and parents pointers, which help during the mining process. On the other hand, a leaf node additionally preserves timestamp information along with all this information. As a result, the tail node inherits an ordinary node's properties; yet, an ordinary node does not represent all of the data that the tail node represents.

#### 4.1.2 Structure of R3PStreamSW-List

R3PStreamSW-List maintains four fields related to an item *i* in the current window *TSW*: support of item *i*–*SupTSW (i), PSTSW (i)*–presents periodic support of item *i* and previous timestamp of item *i*–*TLast (i)*. Along with the R3PStreamSW-Tree, R3PStreamSW-List structure is simultaneously created, which helps in the computation of periodic support of itemsets.

#### 4.1.3 Structure of R3PStreamSW-Queue

In order to make the R3PStreamSW-Tree ready-to-mine, the current window information is captured in the R3PStreamSW-Tree. As the new batch of streams arrives, the oldest batch of streams is deleted to make room for new transactions. To accomplish this task, R3PStreamSW-Growth uses a queue structure named R3PStreamSW-Queue. R3PStreamSW-Queue is a linear data structure that points to the leaf nodes of R3PStreamSW-Tree that hold the timestamp information.

### 4.2 Construction phase of R3PStreamSW-Growth

The construction phase of R3PStreamSW-Growth is a two-step process, and is mentioned in Algorithm 1. At first, the initial window is created, which captures the first set of high-speed stream data. Next, the window sliding phase is accomplished, which is responsible for deleting the old batch of stream data followed by capturing the new set of data streams in the window. These two phases collectively keep the R3PStreamSW-Tree in a ready-to-mine state. R3PStreamSW-Growth accepts stream data *TDS* along with periodicity threshold *maxPer* and periodic support thresholds *minFreqPS* and *minRarePS*. In order to maintain the sliding window, window size– *TSWindowSize* and batch size–*TSBatchSize* values are accepted by the user. R3PStreamSW-Growth algorithm initially asks the user to select any canonical order (*CO*) such as alphabetical order or specific order based on dataset properties and the R3PStreamSW-Tree is built using this order.

**Algorithm 1:**
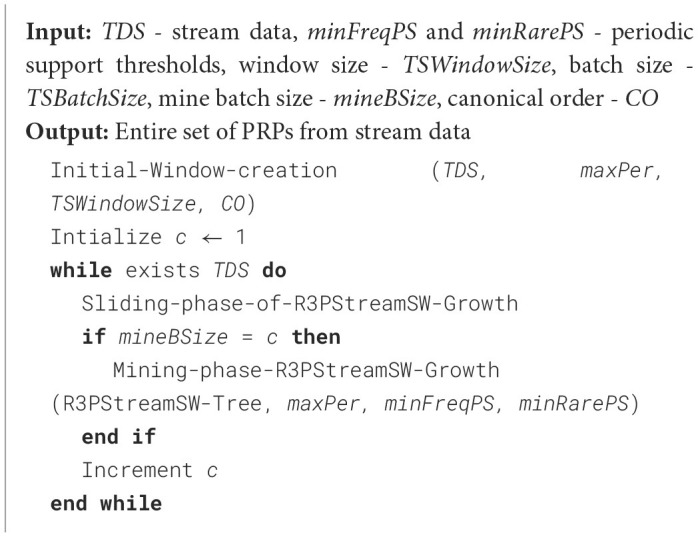
Construction and mining phase of R3PStreamSW-Growth.

#### 4.2.1 Initial window creation phase of R3PStreamSW-Growth

Algorithm 2 presents the initial window creation process. During the phase, R3PStreamSW-Tree captures *TSWindowSize* number of stream transactions in a single scan. As shown in line 1, initially, the root-node of the R3PStreamSW-Tree is created with the label “NULL”. The transaction id, *tid*_*cur*_, is assumed to have continuous values starting from 1. Line 3 ensures *TSWindowSize* number of transactions are captured into the R3PStreamSW-Tree and accordingly, the R3PStreamSW-List and R3PStreamSW-Queue are simultaneously created. The sort order *CO* specified by the user is maintained for every transaction, as depicted in line 4. Lines 5 to 17 present how periodic support of every item in the transaction of the current window is updated in R3PStreamSW-List. If the item “*i”* is appearing for the first time in a window, then “*i”* is added into R3PStreamSW-List. The support *SupTSW (i)* and periodic support *PSTSW (i)* of *i* are initialized to a value “1” and “0”, respectively. Otherwise, the support *SupTSW (i)* of *i* is incremented by 1. Further, the current inter-arrival time of *i* is computed by the difference between the previous tiwmestamp of item *i*–*TLast (i)* and the timestamp of the current transaction *tsw*_*cur*_. If the resultant inter-arrival time satisfies the *maxPer* threshold, then the periodic support *PSTSW (i)* of item *i* is incremented. Before considering the next item, *TLast (i)* is updated with *tsw*_*cur*_, which helps in the computation of the next inter-arrival time.

**Algorithm 2:**
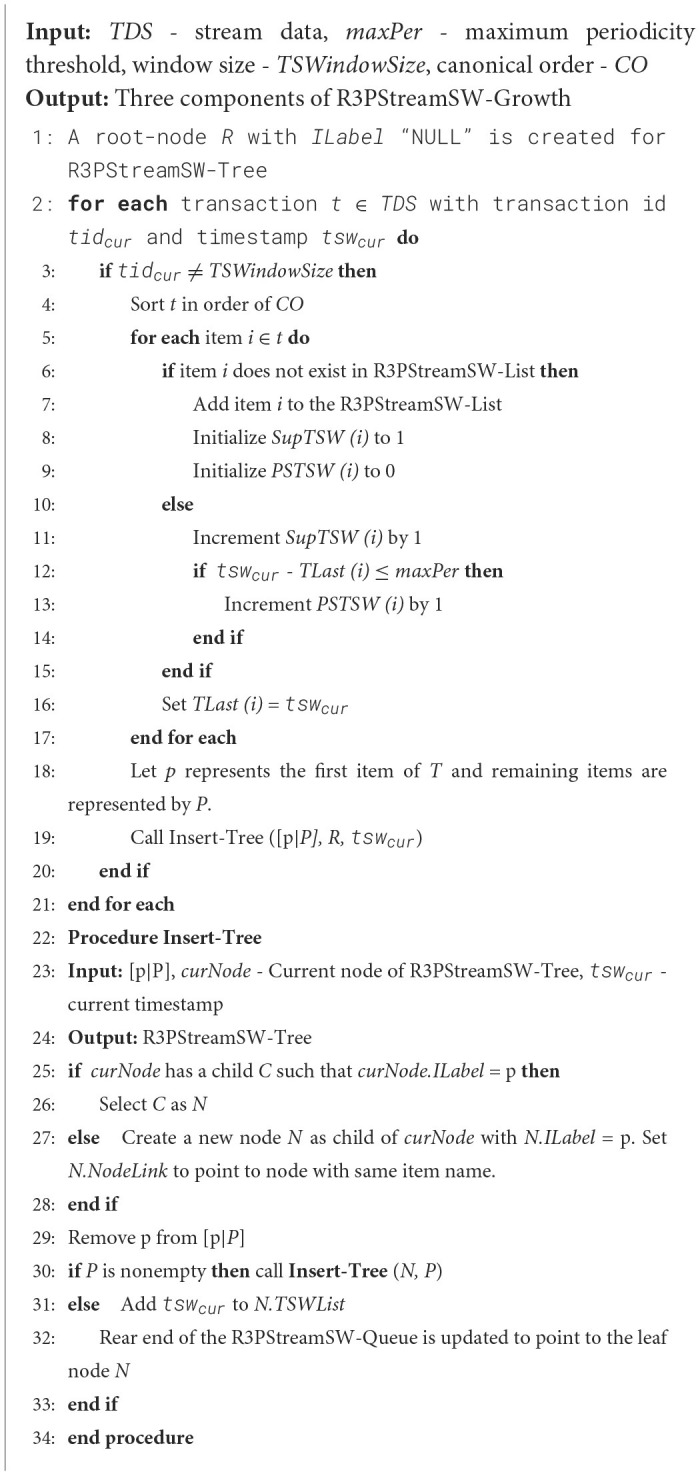
Initial-window-creation.

#### 4.2.2 Insertion of a transaction into R3PStreamSW-Tree

Once R3PStreamSW-List is updated with all the items of the current transaction, then the *Insert-Tree* procedure is called to insert the current transaction into R3PStreamSW-Tree. The items are inserted into R3PStreamSW-Tree similar to *3P-tree*. If R3PStreamSW-Tree contains a similar path for transaction *t*, then the path is shared by adding the remaining path (if any) of *t* at the end. On the other hand, if R3PStreamSW-Tree has no common path, then transaction *t* is added as a new path. The current timestamp information *tsw*_*cur*_ is added to the *TSWList* of the tail node *N*. Simultaneously, the R3PStreamSW-Queue is modified such that the new leaf node *N* is pointed by the rear end. Further, in a similar manner, *TSWindowSize* sized stream transactions are captured into the R3PStreamSW-Tree.

With respect to the sample data stream given in Table 1, Figure 1 depict how the stream transactions are captured into R3PStreamSW-List and R3PStreamSW-Tree with *TSWindowSize* considered as 5. Here, lexicographic order is used to build the R3PStreamSW-Tree. Let *minFreqPS* and *minRarePS* be considered as 3 and 2 respectively. Initially, the itemsets of the first stream transaction *m, n*, and *o* are inserted into the R3PStreamSW-List. The *SupTSW* of all the inserted items are initialized with a value “1”. As the items are appearing for the first time, the *PSTSW* values of these items are set to “0”. The *TLast* of all the inserted items are set with the current timestamp value “1”. Once the R3PStreamSW-List updation is completed, then the current transaction is inserted into the R3PStreamSW-Tree as shown in Figure 1a. As there is no common path in the R3PStreamSW-Tree, a new path is created with the node *m* becoming a child node for the root. Then nodes *n* and *o* are added to this path. As node *o* is the tail node, the timestamp information “1” is added to its *TSWList*. Now, the R3PStreamSW-Queue is updated, and a new pointer is added from the rear end, which is pointing to the current tail node *o*. Figure 1b shows the scanning and insertion process of transaction “T2”. Similar to the first transaction, the items *q, r* and *s* are inserted into R3PStreamSW-List. Further, a new path is constructed in the R3PStreamSW-Tree and the current timestamp information “3” is added to the tail-node *s*. The R3PStreamSW-Queue is modified as a new pointer is added which is pointing to the current tail node *s*. Figure 1c shows how the periodic support is changed for the items *m, n, o* and *r* after scanning the third transaction. The *TLast* of item *m* is “1” and the current timestamp information “3”, hence the inter-arrival time resulted as “2”. This value satisfies the *maxPer* threshold and accordingly *PSTSW (m)* is incremented. In a similar fashion, the periodic support *PSTSW* value of the items is incremented if the inter-arrival time satisfies *maxPer* threshold. Next, as there is a common path for transaction “T3”, the path is shared in the R3PStreamSW-Tree as shown in Figure 1c. Since the item *r* does not exist in the path, it is attached at the end, and the timestamp information “3” is added to the *TSWList*. Now, the R3PStreamSW-Queue is modified, and a new pointer is added, which is pointing to the current tail node *r*. It can be noted that R3PStreamSW-Queue successfully maintains the duplicate timestamp information. This helps in the window sliding phase to delete the duplicate occurrences in the order of their insertion. Figure 1d shows the updated R3PStreamSW-List and R3PStreamSW-Tree after insertion of “T4”. Figure 1e shows the resultant R3PStreamSW-List and R3PStreamSW-Tree after capturing the initial window *TSWindowSize* i.e., five transactions in the stream data. Similar to FP-Tree, different pointers are maintained in R3PStreamSW-Tree, and for clarity, it is not shown in Figure 1. The resultant R3PStreamSW-Tree shown in Figure 1e represents the compact and complete initial window stream timestamp information *TSW* using which entire R3Ps of the current window can be extracted. The completeness of R3PStreamSW-Tree can be defined with the following property and lemma. Let for every transaction *t* in the stream timestamp window *TSW*, all the items in *t* are represented as *item (t)* which present complete projection of *t*.

**Property 6:** In current timestamp window *TSW, item (t)* of each transaction *t* is maintained only once in the R3PStreamSW-Tree. Further, *i*_*Tail*_ representing the tail node of this path stores the timestamp *tsw*_*cur*_ only once.

**Lemma 4.2**. For current timestamp window *TSW* of the stream dataset *TDS, item (t)* of each transaction *t* can be extracted from R3PStreamSW-Tree.

**Proof** According to Property 6, *item (t)* of every transaction *t* in current timestamp window *TSW* is mapped to R3PStreamSW-Tree at best by a single path and any path starting from the root up to the leaf node *i*_*Tail*_ holds the complete projection of exactly *x* transactions, where *x* is the total count of timestamp information maintained in *TSWList* field of *i*_*Tail*_.

The total size contribution of all transactions in current timestamp window *TSW* can be at best by


$$\begin{array}{l} \underset{t\in TSW}{\sum }|Size\left(t\right)|. \end{array}$$

As there are multiple paths that are shared in the R3PStreamSW-Tree, therefore the size of R3PStreamSW-Tree is much smaller than


$$\begin{array}{l} \underset{t\in TSW}{\sum }|Size\left(t\right)|. \end{array}$$

#### 4.2.3 Sliding window phase of R3PStreamSW-Growth

R3PStreamSW-Growth efficiently incorporates sliding window phase by (i) Removing the oldest *TSBatchSize* number of transactions from R3PStreamSW-Tree (ii) Scanning the recent *TSBatchSize* number of transactions from the stream and inserting the transactions into R3PStreamSW-Tree (iii) Updating the support and periodic support information by refreshing the R3PStreamSW-List according to the updated R3PStreamSW-Tree. Here, R3PStreamSW-Queue plays an important role which holds the pointers to every tail node in the R3PStreamSW-Tree and avoids traversing the entire R3PStreamSW-Tree during the window sliding phase.

##### 4.2.3.1 Removal of oldest transactions from R3PStreamSW-Tree

To efficiently handle the removal of the oldest batch of transactions, an alternative approach without incurring the expense of tree traversal is used and is given in Algorithm 3. The algorithm starts with traversing the tail nodes pointed by R3PStreamSW-Queue and then going up to the root node of the tree, satisfying the conditions as shown in Algorithm 3. Instead of navigating the entire R3PStreamSW-Tree, only the tail nodes of the expired transactions are reached by traversing from the front end of the R3PStreamSW-Queue. Further, *TSWList* of these tail nodes are solely modified to reflect the deletion of transactions as depicted in line 18 of Algorithm 3. The process involves the deletion of time stamps from the *TSWList* of each tail node. During this deletion process, if a tail-node's *TSWList* becomes empty, then the removal process of both the tail-node and its path leading up to the root node happens, as shown in Algorithm 3 from lines 7 to 15. Further, the nodes are deleted in the tree following the parent node if the parent node does not have any other child apart from the current traversal path. This approach ensures that only the transactions that have expired are removed from the tree. R3PStreamSW-Queue is updated by removing the tail node pointers of the deleted batch of transactions, as shown in line 4 of Algorithm 3. The deletion process ends when *TSBatchSize* number of expired transactions have been traversed. Finally, both R3PStreamSW-Tree and R3PStreamSW-Queue are ready for insertion of a new batch of stream data.

**Algorithm 3:**
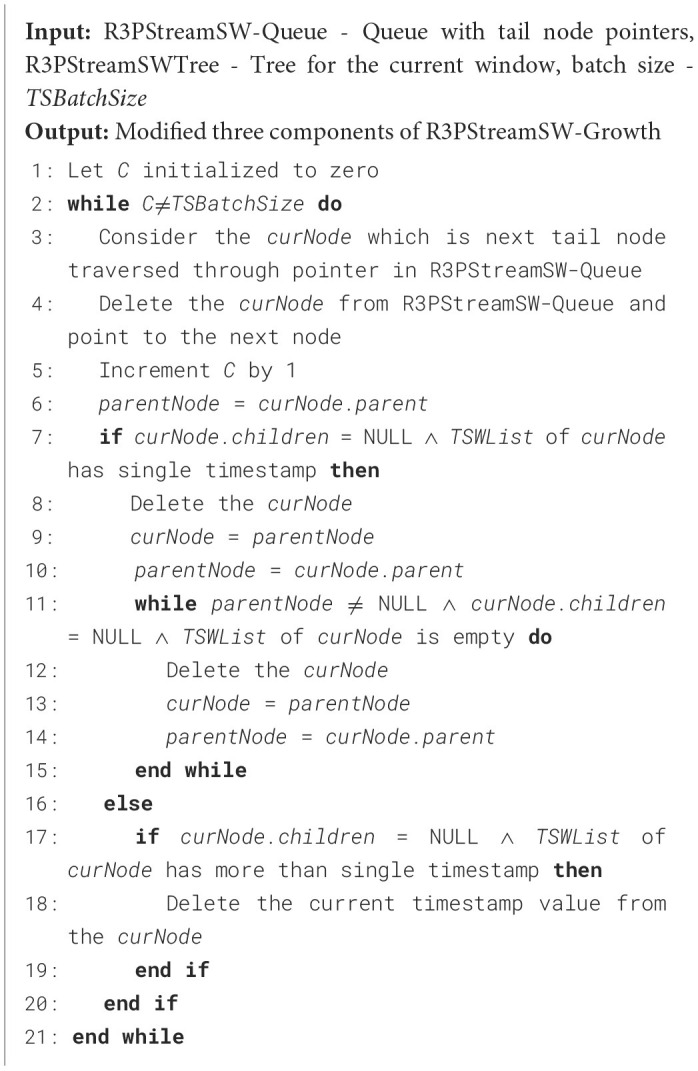
Removal-of-oldest-bach.

**Figure 2:**
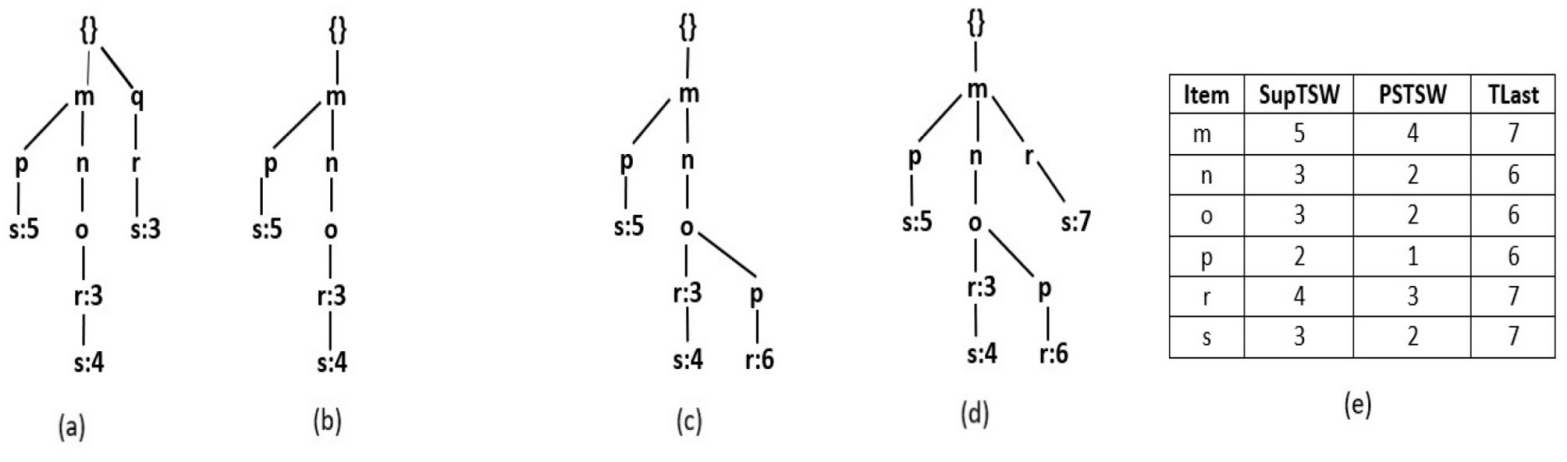
Resultant R3PStreamSW-Tree: **(a)** After deletion of T1. **(b)** After deletion of T2. **(c)** After insertion of T3. **(d)** After insertion of T4. **(e)** Resultant R3PStreamSW-List after the first window sliding phase.

Figure 2a depicts the deletion of the oldest transaction with the timestamp value “1”. As the first tail-node with item “*o”* is also shared by another path, only the timestamp information is deleted from its *TSWList*. Further, even though there are multiple transactions with timestamp ‘3', R3PStreamSW-Queue points to node with *ILabel* “s”, which is the oldest transaction. After the timestamp information is removed from the *TSWList* of node “*s”*, the node itself is deleted as it is not shared by any other path and its *TSWList* is also empty. Further, as shown in Figure 2b, the entire path is removed as there are no multiple paths for any other node in that path. As batch size *TSBatchSize* is considered as 2, the removal process of expired transactions in the stream is completed, and now the R3PStreamSW-Tree is ready for the insertion phase.

##### 4.2.3.2 Insertion of a new batch of transactions into R3PStreamSW-Tree

The process of inserting a new set of *TSBatchSize* number of transactions into the R3PStreamSW-Tree is shown in Figures 2c, d. The tree creation process and R3PStreamSW-Queue updation process is similar to that followed in the initial window tree creation phase.

##### 4.2.3.3 Refreshing the R3PStreamSW-List

During the window sliding phase, it is possible that periodic patterns can become aperiodic and vice versa. Therefore, the R3PStreamSW-List also needs to be updated according to the timestamp information represented by the resultant R3PStreamSW-Tree shown in Figure 2d. This is called refreshing the R3PStreamSW-List and is presented in Figure 2e. It can be seen in Figure 2e that the item *q* is removed as its support is “0”.

### 4.3 Mining phase of R3PStreamSW-Growth

As R3PStreamSW-Tree is a novel tree structure which captures the complete timestamp information of sliding window *TSW* in a single scan, similar to 3P-Growth, a pattern-growth bottom-up approach is used to mine entire set of R3Ps. As shown in Algorithm 1, the value of *mineBSize* is accepted, which decides after how many batches of sliding window process the mining happens. The following important property and lemma are defined to mine the R3PStreamSW-Tree recursively:

**Property 7**: Every tail node in a R3PStreamSW-Tree maintains the timestamp information of all the nodes in the path (from tail node up toward the root node) in its *TSWList*.

**Lemma 4.3**. Let R3PStreamSW-Tree contains a path V = {*i*_1_,*i*_2_,….,*i*_*Tail*_}, then the *TSWList* field denotes all the timestamp information of the transactions in the current window *TSW* in which *i*_*Tail*_ is the tail node. If the timestamp information from *TSWList* is pushed up to node *i*_*Tail*−1_, then *i*_*Tail*−1_ represents the timestamp information of the path V' = {*i*_1_,*i*_2_,….,*i*_*Tail*−1_} for same set of transactions in the *TSWList* without losing any timestamp information.

**Proof**: The *TSWList* of tail node *i*_*Tail*_ maintains the timestamp information of V' for the same set of transactions. Hence, the same timestamp information for the path V' is maintained by *i*_*Tail*−1_ without losing any timestamp information.

**Figure 3:**
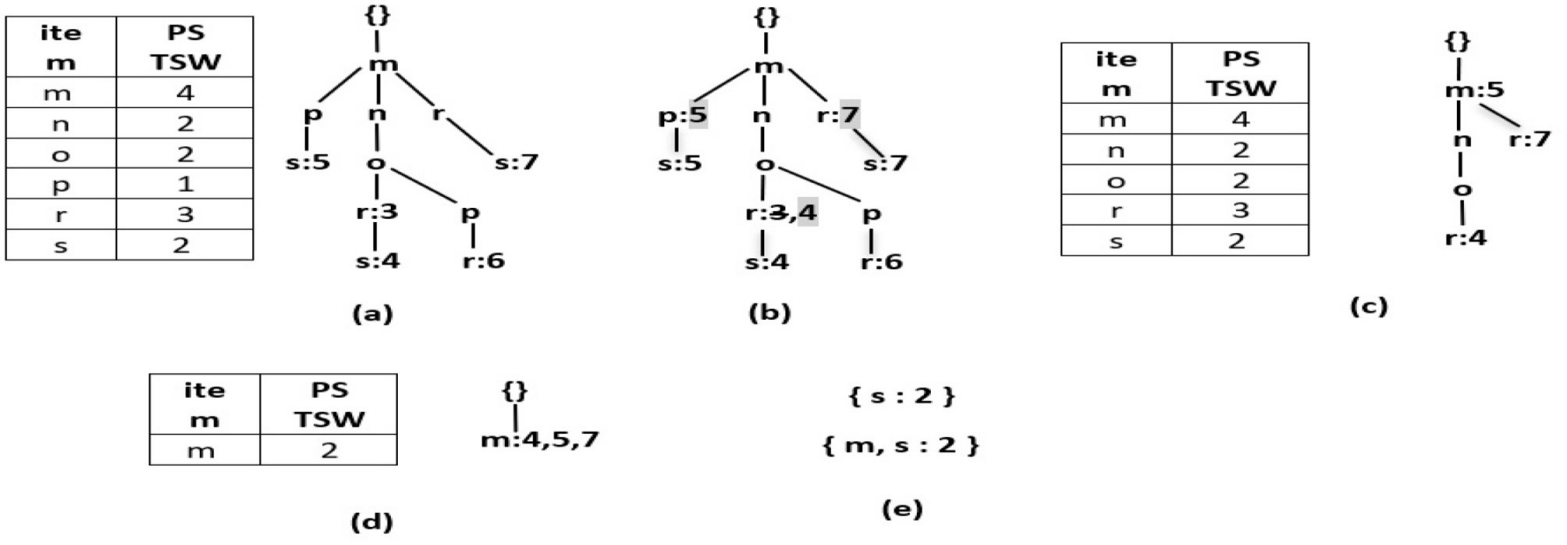
Resultant R3PStreamSW-Tree created during mining of second window of Table 1. **(a)** Initial R3PStreamSW-Tree representing second window. **(b)** Pushing timestamps of *TSWList* to parent nodes. **(c)** Prefix tree for suffix item “s”. **(d)** Conditional tree for suffix item “s”. **(e)** R3Ps generated for suffix item “s”.

The mining algorithm is presented in Algorithm 4. The extraction of the R3PStreamSW-Tree is a three-step process: (i) Initially, each partial periodic item is labeled as the starting suffix pattern. (ii) Following that, a conditional pattern base is established. This base will comprise collections of prefix paths within the R3PStreamSW-Tree that co-occurred with the above suffix patterns. (iii) The subsequent phase involved in the construction of a conditional R3PStreamSW-Tree called *CTSWTree* derived from this conditional pattern base by removing all non-periodic items. This tree is created to facilitate recursive mining. (iv) Ultimately, the generated suffix patterns obtained from the conditional R3PStreamSW-Tree are joined with the original patterns. This amalgamation process resulted in the generation of R3Ps as the final output of the mining process. The operation of Algorithm 4 proceeds as follows: For each partial periodic item present in the R3PStreamSW-List, the conditional pattern base, often referred to as the prefix tree, is constructed. At first, the bottom-most item “*s”* is considered. The prefix sub-paths associated with node “*s”* are collected and organized into a tree structure called *R3PStreamPTs*, which served as the foundation for constructing the prefix tree specific to “*s”*. Since “*s”* occupied the lowest position in the R3PStreamSW-List, every node within the R3PStreamSW-Tree labeled as “*s”* has to be a terminal node. In accordance with Property 7, timestamp lists *TSWList* of each node belonging to “*s”* are explicitly mapped onto all items along the corresponding path within a temporary array *TSW* as shown in line 2 of Algorithm 4, with one array for each item, facilitating the creation of *R3PStreamPTs*. This temporary array significantly simplified the computation of period support for every item in *R3PStreamPTs*. For instance, when item “*X”* within *R3PStreamPTs* met the condition (PSTSW (P) < *minFreqPS* ∧ PSTSW (P) ≥ *minRarePS)*, a conditional tree is built for it and proceeded recursively to extract it in search of partial periodic patterns, as detailed from lines 3 to 5 of Algorithm 4. The timestamp lists *TSWList* were propagated upwards to their respective parent nodes within *R3PStreamPTs*. This step facilitated prefix tree construction for the subsequent item in the R3PStreamSW-List. During mining, the periodic item may be easily determined by performing an O (1) look-up at the *R3PStreamSW-List*, even though the items are not arranged in the R3PStreamSW-Tree according to the support count. The resultant R3PStreamSW-Tree created after each step of the mining phase is shown in Figure 3. A similar approach is repeated for all the items in the R3PStreamSW-List.

**Algorithm 4:**
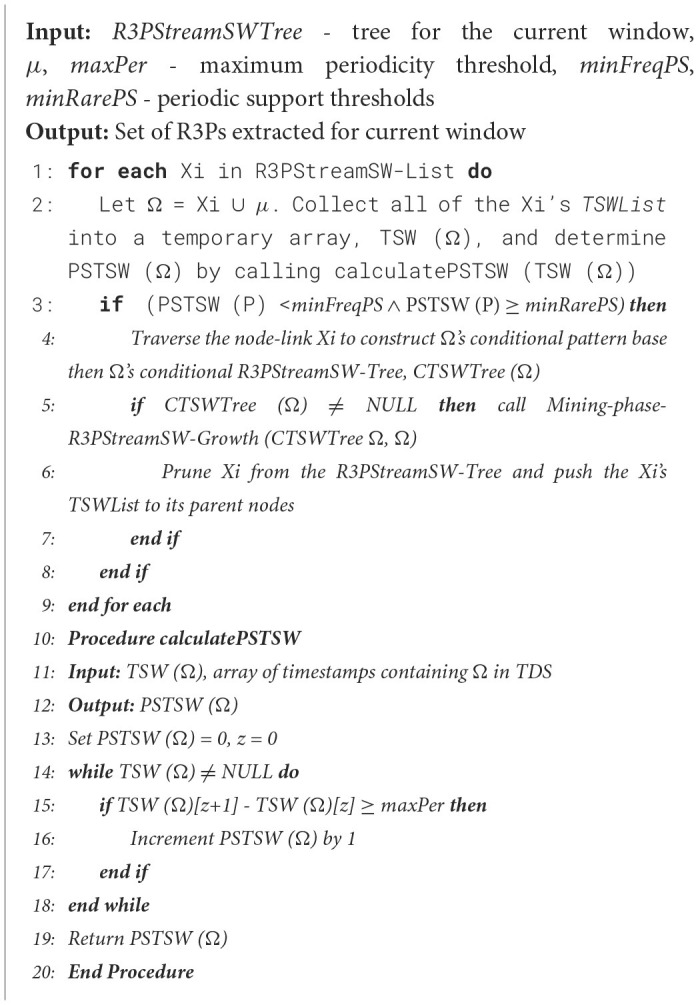
Mining-phase-R3PStreamSW-Growth.

## 5 Rare partial periodic pattern stream sliding window bit vector miner: R3P-StreamSWBitVectorMiner—A list-based framework

During the mining phase, R3PStreamSW-Growth employs a divide-and-conquer strategy, which generates a massive number of conditional pattern trees. This recursive process affects the mining performance. To overcome this, R3P-BitVectorMiner (Upadhya et al., 2023) is enhanced and a novel depth-first search framework named R3P-StreamSWBitVectorMiner is proposed to extract entire R3Ps from a temporal stream data. Here, the number of cyclic repetitions is counted and based upon the user-specified periodic support measures called *minFreqPS* and *minRarePS* the R3Ps are selected. The overall process of R3P-StreamSWBitVectorMiner is presented in Algorithm 5. The framework is divided into three phases: (i) Initial window creation phase (ii) Sliding window phase where old batch of transactions are removed for giving place to new batch of transactions (iii) Mining R3Ps from current stream window.

**Algorithm 5:**
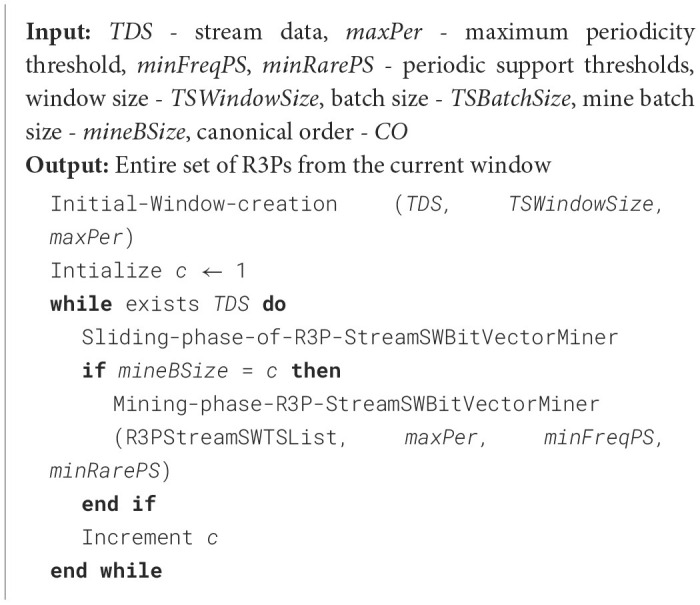
R3P-StreamSWBitVectorMiner.

### 5.1 Initial window creation phase of R3P-StreamSWBitVectorMiner

**Figure 4:**
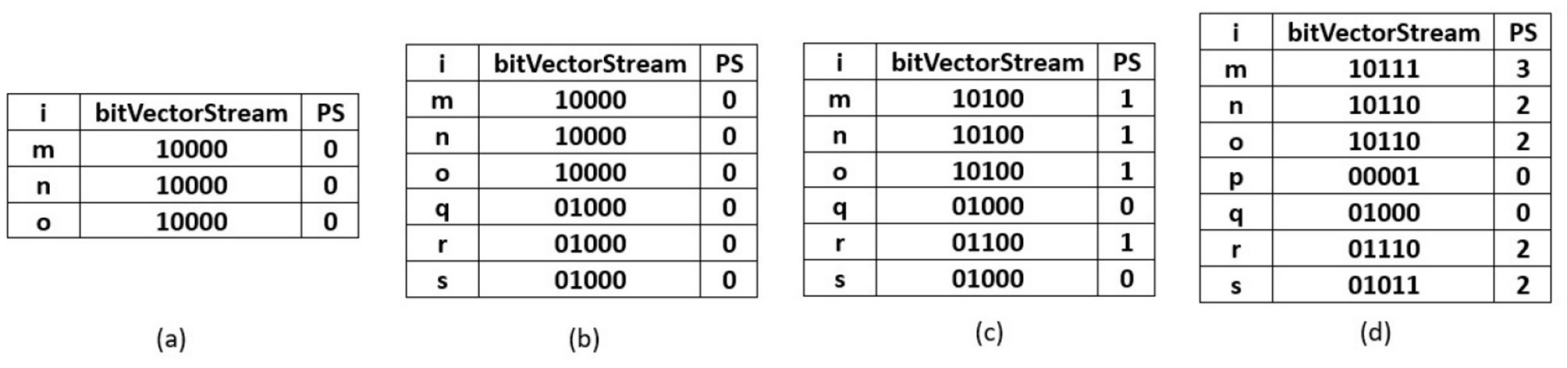
R3PStreamSWTSList: **(a)** After scanning tid = 1. **(b)** After scanning tid = 2. **(c)** After scanning tid = 3. **(d)** After scanning all transactions first window with *TSWindowSize* 5.

In this section, *TSWindowSize* number of initial transactions are captured as presented in Algorithm 6 and it is similar to 3P-BitVectorMiner. The transactions are converted into a bit-vector form and are maintained in a R3PStreamSWTSList structure, which is similar to 3PTSList. Each bit in the bit-vector represents consecutive temporal transactions where the presence of an item is indicated by “1” and absence by “0”. As every transaction is transformed into a bit-vector form, the R3PStreamSWTSList structure is updated for all the items appearing in the transactions. Along with the modification of the bit-vector, the periodic support values are updated in the R3PStreamSWTSList as shown in Algorithm 6. As shown in line 3 of Algorithm 6, the current time stamp information *ts*_*cur*_ is stored in an array *TSStreamList* and it is used as common time stamp information for all the items. Lines 9 to 10 of Algorithm 6 show how the time stamp value can be acquired by extracting the information from the *TSStreamList* array for the required bit of *bitVector (i)*. As observed in line 11 of Algorithm 6, the current periodicity is computed by subtracting the current time stamp from the previous time stamp obtained from array *TSStreamList*. The periodic support *PSTSW* value of the current item *i* is incremented by one if the resultant periodicity value is not greater than *maxPer* threshold. Table 1 presents temporal transactions in a data stream *TDS*. Let the window size *TSWindowSize* and batch size *TSBatchSize* be 5 and 2 respectively. The sequence of bit-vector representation after scanning of various transactions in the initial window creation of R3P-StreamSWBitVectorMiner is shown in Figure 4.

**Algorithm 6:**
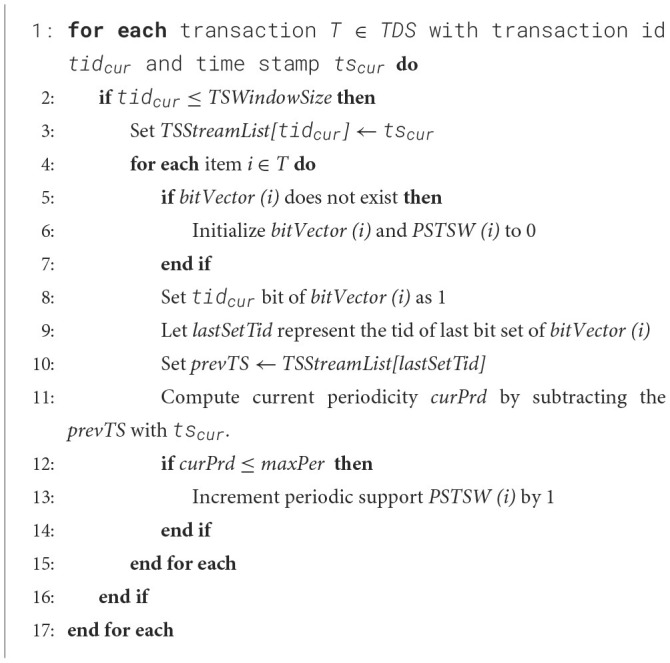
Initial-Window-creation (*TDS* - stream data, *TSWindowSize* - window size, *maxPer* - maximum periodicity threshold).

### 5.2 Sliding window phase of R3P-StreamSWBitVectorMiner

**Figure 5:**
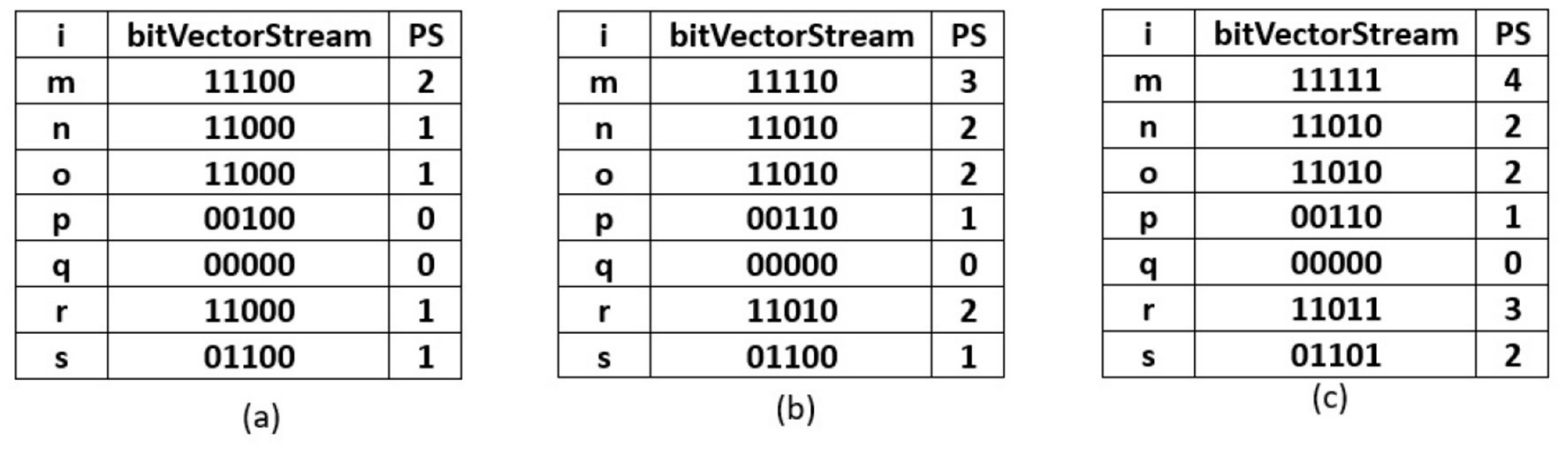
R3PStreamSWList: **(a)** After removal of *TSBatchSize* number of transactions. **(b)** After scanning tid = 6. **(c)** After scanning tid = 7.

Once *TSWindowSize* transactions are scanned, the current window becomes full. Further, the sliding window phase begins where the initial removal of the oldest batch of transactions happens. The removal process is carried out by performing a left shift bit-wise operation on the items present in the R3PStreamSWTSList. The left shiting operation removes *TSBatchSize* number of transactions from R3PStreamSWTSList as shown in Line 2 of Algorithm 7. Further, the same number of *TSBatchSize* recent transactions are scanned from input stream *TDS* and are inserted into the R3PStreamSWTSList. The insertion process is similar to the initial-window-creation process. As the *TSBatchSize* is 2, the resultant window after removing the oldest two transactions is shown in Figure 5a. Next, transaction with tid = 6 is inserted as shown in Figure 5b, and the resultant window after the sliding phase is shown in Figure 5c.

**Algorithm 7:**
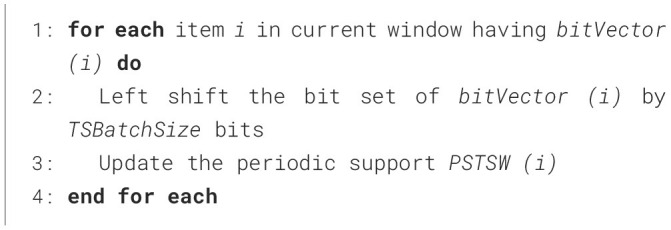
Remove-Oldest-Batch-R3P-StreamSWBitVectorMiner (R3PStream SWTSList - List Structure, *maxPer* - maximum periodicity threshold, batch size - *TSBatchSize*).

### 5.3 Mining phase of R3P-StreamSWBitVectorMiner

**Figure 6:**
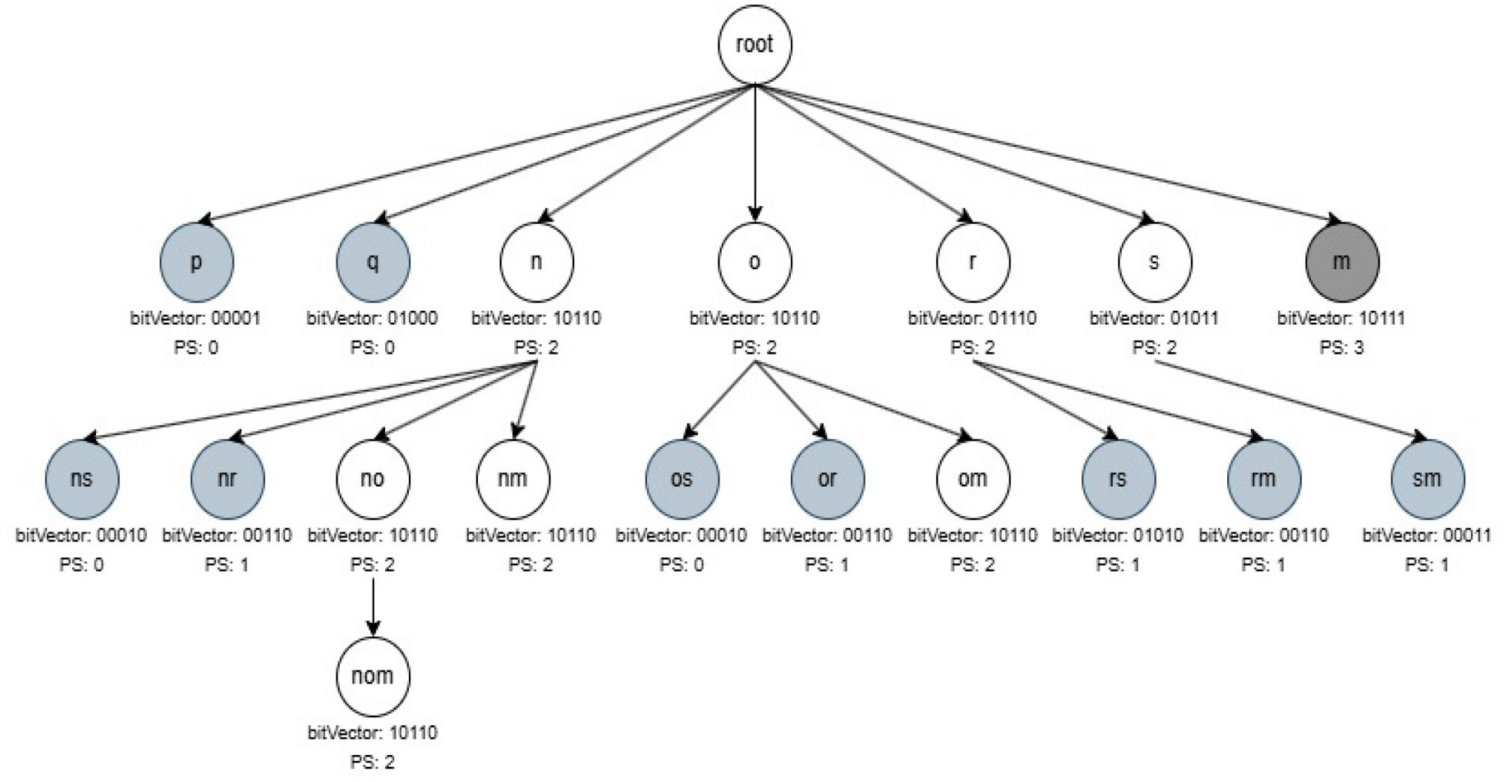
Resultant R3Ps after mining first window.

**Figure 7:**
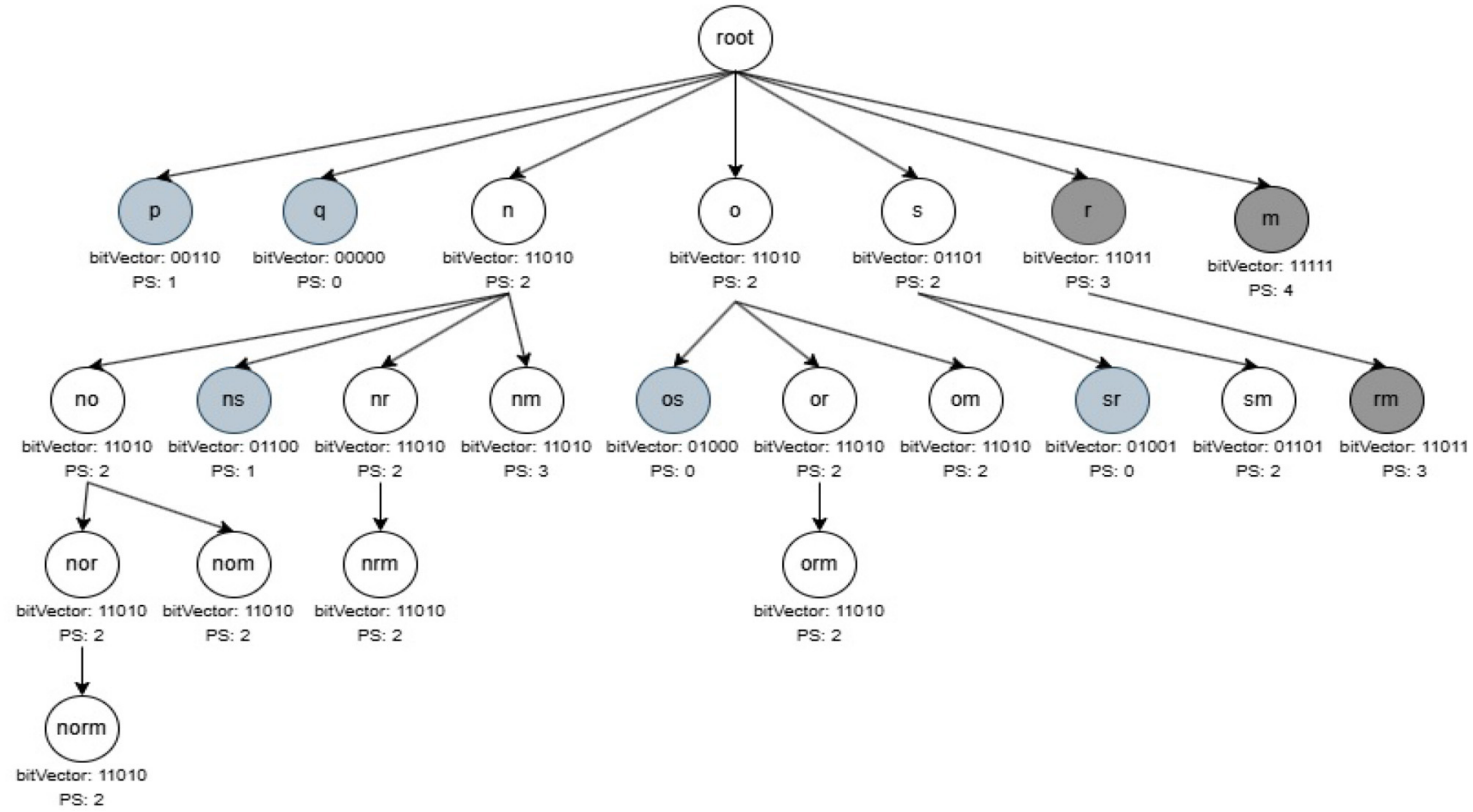
Resultant R3Ps after mining second window.

During the mining phase, initially all non-periodic and noisy one-length itemsets from R3PStreamSWTSList are removed. Further, Constructing R3PStreamSWTSTree and recursively traversing R3PStreamSWTSTree in Depth First Search (DFS) method to extract complete set of R3Ps by discarding non-periodic and noisy patterns is similar to the process of mining phase of 3P-BitVectorMiner (Upadhya et al., 2023). Let *minFreqPS* and *minRarePS* be considered as 3 and 2 respectively. The resultant R3PStreamSWTSTree after the DFS traversal of all items from the first window is presented in Figure 6. By Definition 3.4 all the pattern “i” with (*PSTSW (i)* = 2) represented in white color are selected as output R3Ps. Whereas, the pattern “i” with (*PSTSW (i)* < 2) represented in light gray color are treated as noisy itemsets. The path of the noisy itemsets are not continued further which reduce the search space during mining process. Moreover, the dark gray-represents pattern “i” with (*PSTSW (i)* ≥ 3) is a frequent pattern. Even though the frequent patterns are not shown as output, the mining process continues in these paths since their supersets can be rare partial periodic patterns. Figure 7 shows the final resultant R3PStreamSWTSTree following the DFS traversal of all items from second window.

## 6 Experimental analysis

### 6.1 Experimental setup

The proposed frameworks, *R3PStreamSW-Growth* and R3P-StreamSWBitVectorMiner, extract the periodic patterns using *maxPer* threshold value. Further, only the rare patterns from the vast search space are retained with the help of *minFreqPS* and *minRarePS* thresholds. To accomplish the stream data mining, *TSWindowSize*, a value representing different window sizes is used with *TSBatchSize* and *mineBSize*. The values for *TSBatchSize* and *mineBSize* indicate every time *TSBatchSize* transactions are taken into account during the window sliding phase, and the mining process occurs following a *mineBSize* number of sliding phase repetitions. The experiments are conducted in three ways to show the influence of variation in (i) *minRarePS* threshold value (ii) *minFreqPS* threshold value (iii) *maxPer* threshold value. In comparison with frequent periodic patterns, periodic rare patterns are patterns having larger periodicity and low support, various experiments are carried out by setting the threshold values accordingly. The proposed algorithm is evaluated on a system equipped with an Intel (R) Core (TM) i5-7400 CPU with 8GB of RAM operating at 3.00 GHz and running Windows 10 Enterprise. The algorithms are implemented on the Java platform.

### 6.2 Datasets used

The synthetic and real datasets with different transaction sizes used for the experimentation are shown in Table 2. These datasets are downloaded from the repository https://github.com/udayRage/pykit_old/tree/master/Datasets which are frequently used in temporal mining algorithms. *Retail* dataset has 88k transactions, while *Accidents* dataset comprises 340k transactions. *T10I4D100K* is a sparse synthetic dataset produced by the IBM data generator. A tiny real-world *Chess* dataset consists of 3,000 transactions.

**Table 2:** Statistics of datasets.

**S. No**	**Database**	**Type**	**Nature**	**Transaction length**			**Database size**
				**Min**.	**Avg**.	**Max**.	**(In Count)**
1	Accidents	Real	Dense	18	33.8	51	3,40,183
2	T10I4D100K	Synthetic	Sparse	1	10	29	1,00,000
3	Retail	Real	Sparse	2	12	77	88,162
4	Chess	Real	Dense	37	37	37	3,196

### 6.3 Runtime comparison

The runtime performance of the algorithms is determined by carrying out various experiments, taking into consideration numerous datasets for varied minimum support and maximum periodicity threshold values. In addition, the execution time is observed by varying the window and batch size of input stream data.

**Figure 8:**
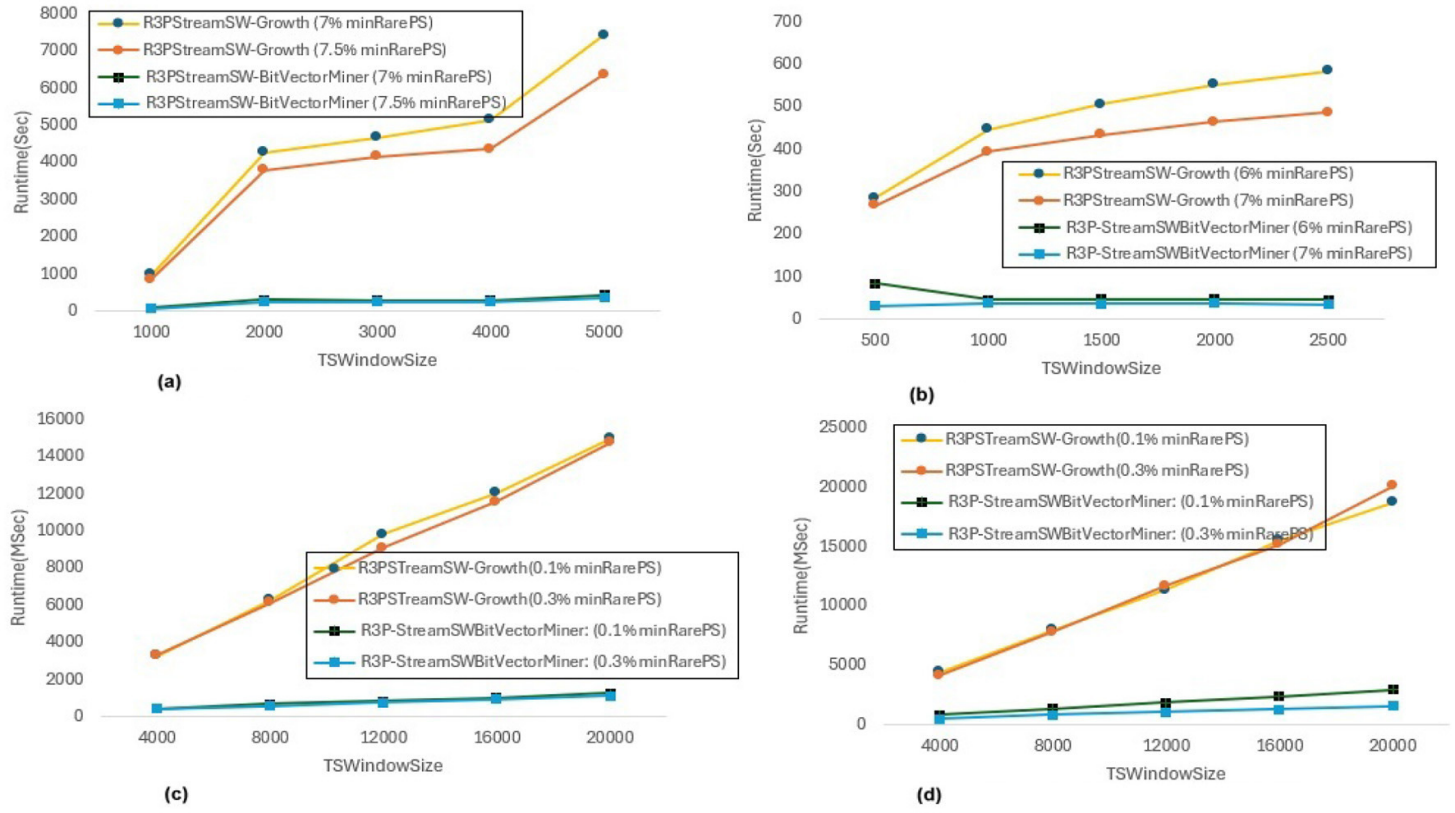
Runtime comparison for *minRarePS* variations. **(a)** Accidents, maxPer = 30%, minFreqPS = 0.8%. **(b)** Chess, maxPer = 30%, minFreqPS = 0.8%. **(c)** T10I4D100K, maxPer = 70%, minFreqPS = 0.5%. **(d)** Retail, maxPer = 70%, minFreqPS = 0.5%.

**Figure 9:**
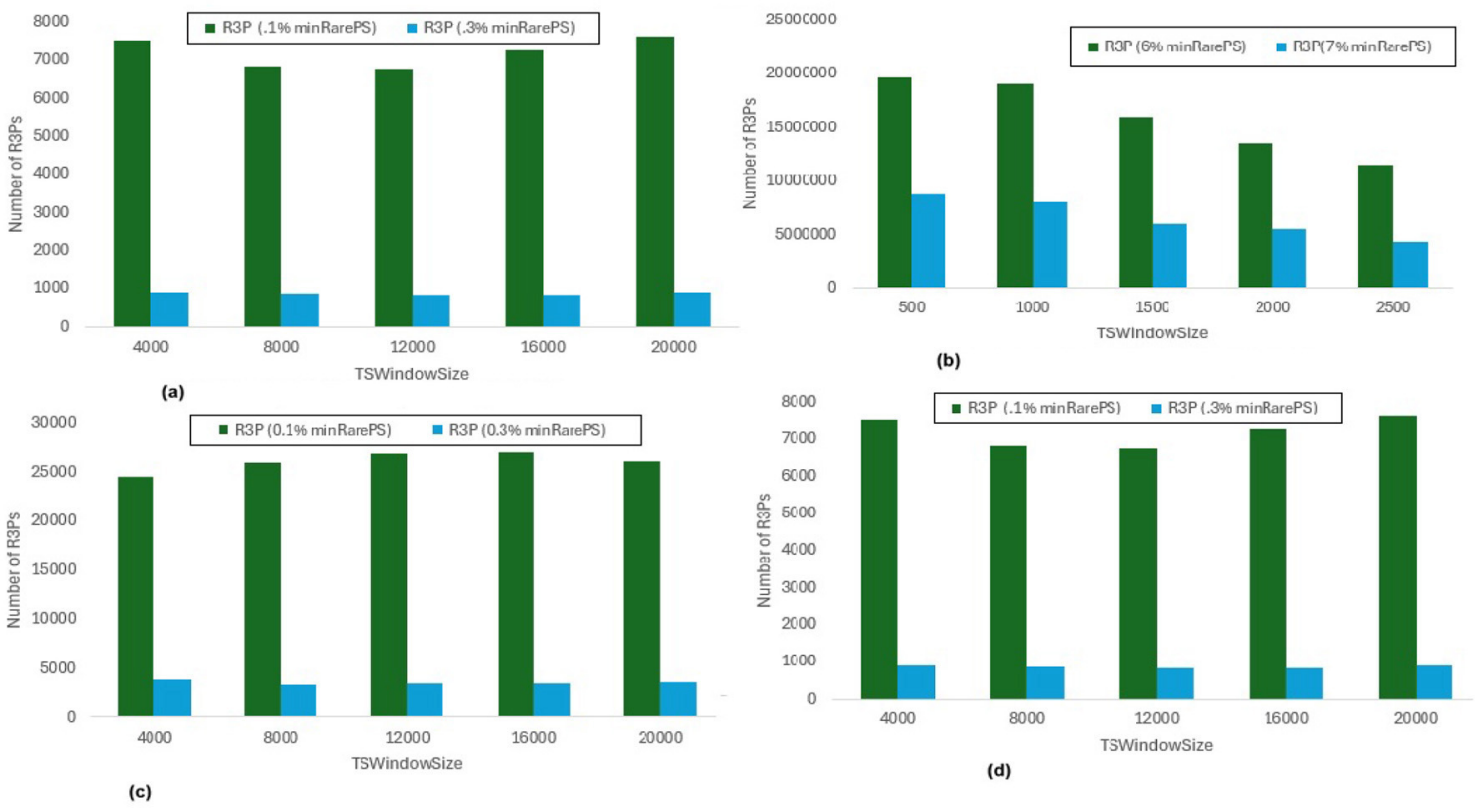
Number of R3Ps generated for *minRarePS* variations. **(a)** Accidents, maxPer = 30%, minFreqPS = 0.8%. **(b)** Chess, maxPer = 30%, minFreqPS = 0.8%. **(c)** T10I4D100K, maxPer = 70%, minFreqPS = 0.5%. **(d)** Retail, maxPer = 70%, minFreqPS = 0.5%.

**Figure 10:**
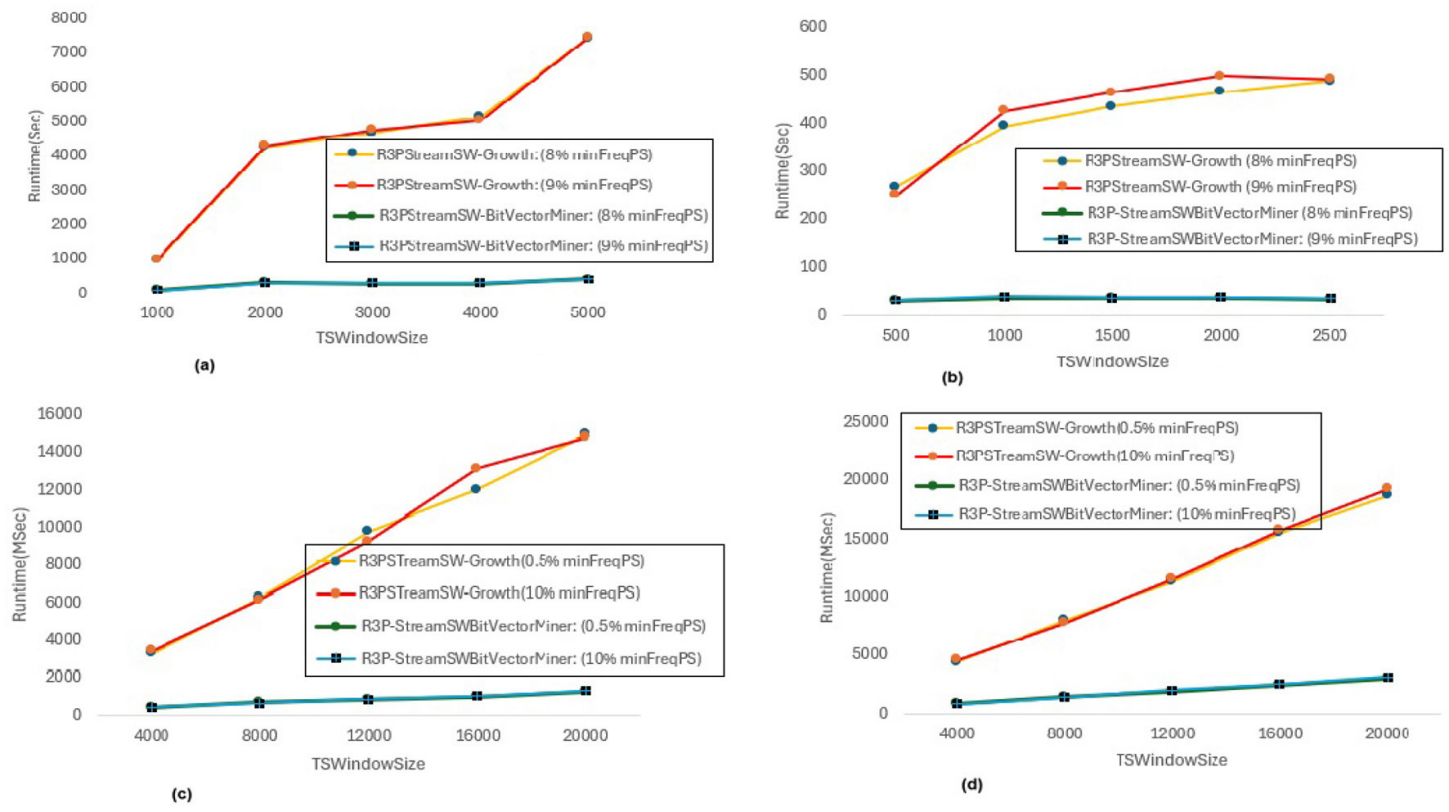
Runtime comparison for *minFreqPS* variations. **(a)** Accidents, maxPer = 30%, minRarePS = 7%. **(b)** Chess, maxPer = 30%, minRarePS = 7%. **(c)** T10I4D100K, maxPer = 70%, minRarePS = 0.1%. **(d)** Retail, maxPer = 70%, minRarePS = 0.1%.

**Figure 11:**
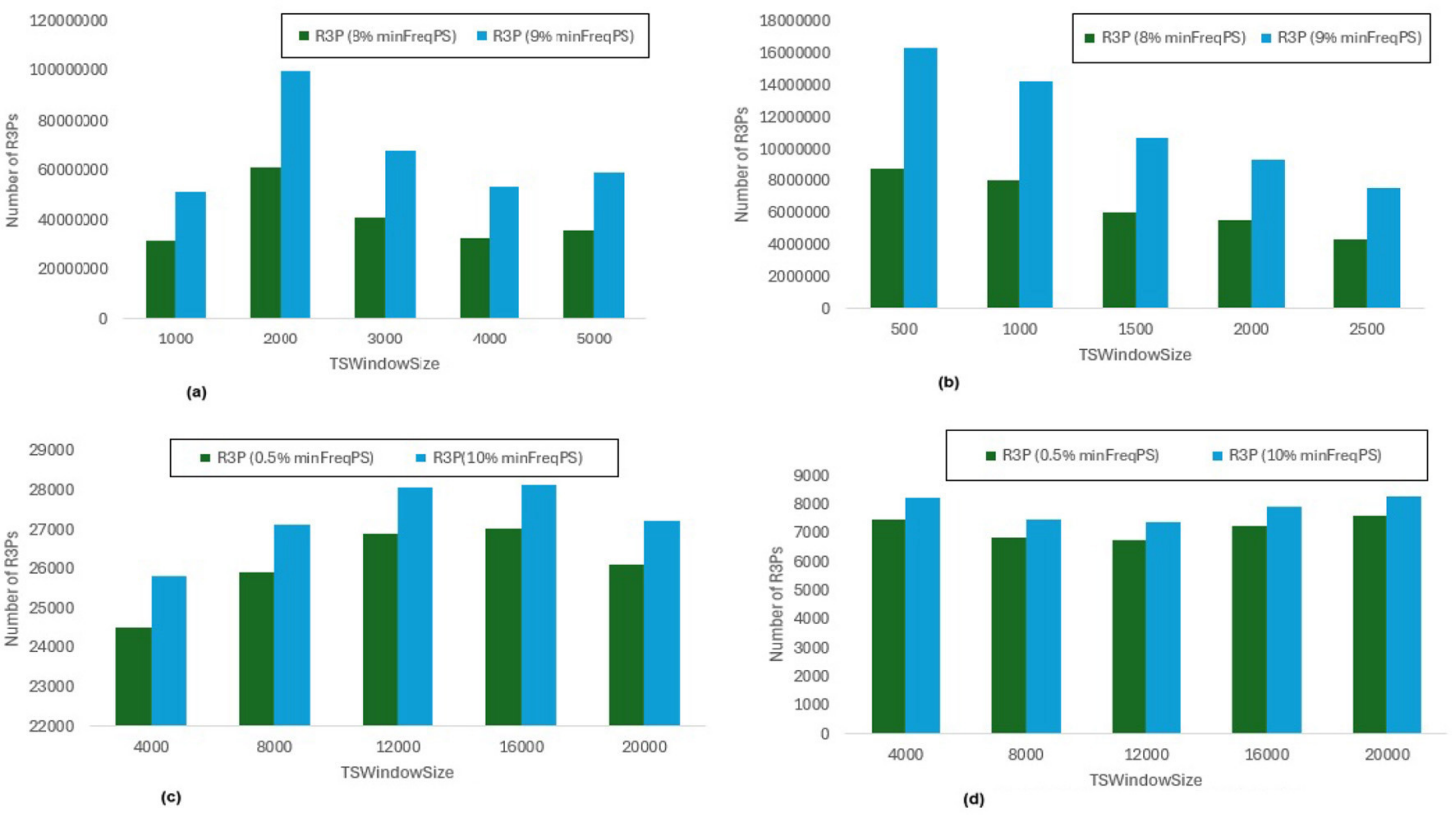
Number of R3Ps generated for *minFreqPS* variations. **(a)** Accidents, maxPer = 30%, minRarePS = 7%. **(b)** Chess, maxPer = 30%, minRarePS = 7%. **(c)** T10I4D100K, maxPer = 70%, minRarePS = 0.1%. **(d)** Retail, maxPer = 70%, minRarePS = 0.1%.

**Figure 12:**
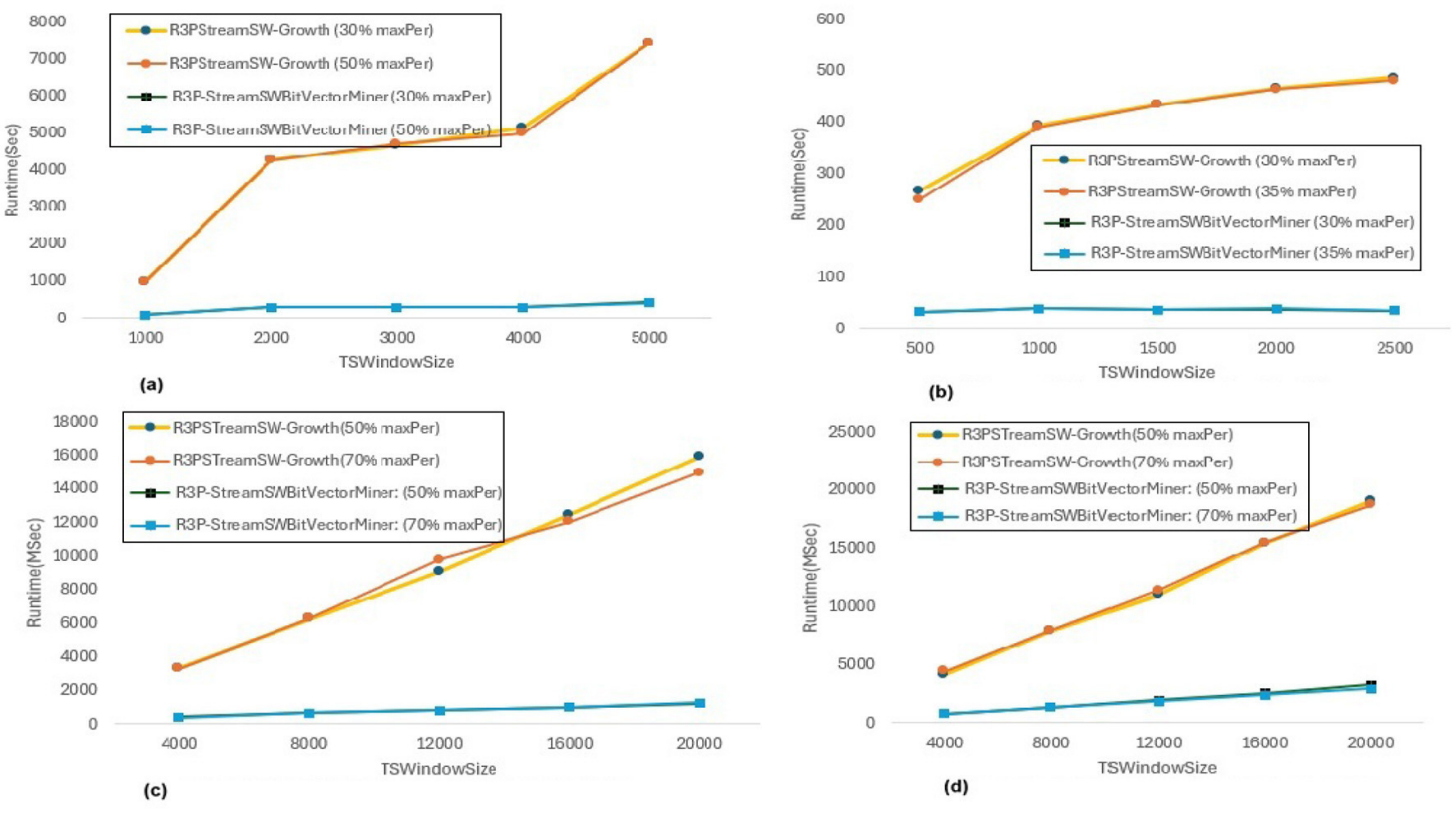
Runtime comparison for *maxPer* variations. **(a)** Accidents, minRarePS = 7%, minFreqPS = 8%. **(b)** Chess, minRarePS = 7%, minFreqPS = 8%. **(c)** T10I4D100K, minRarePS = 0.1%, minFreqPS = 0.5%. **(d)** Retail, minRarePS = 0.1%, minFreqPS = 0.5%.

**Figure 13:**
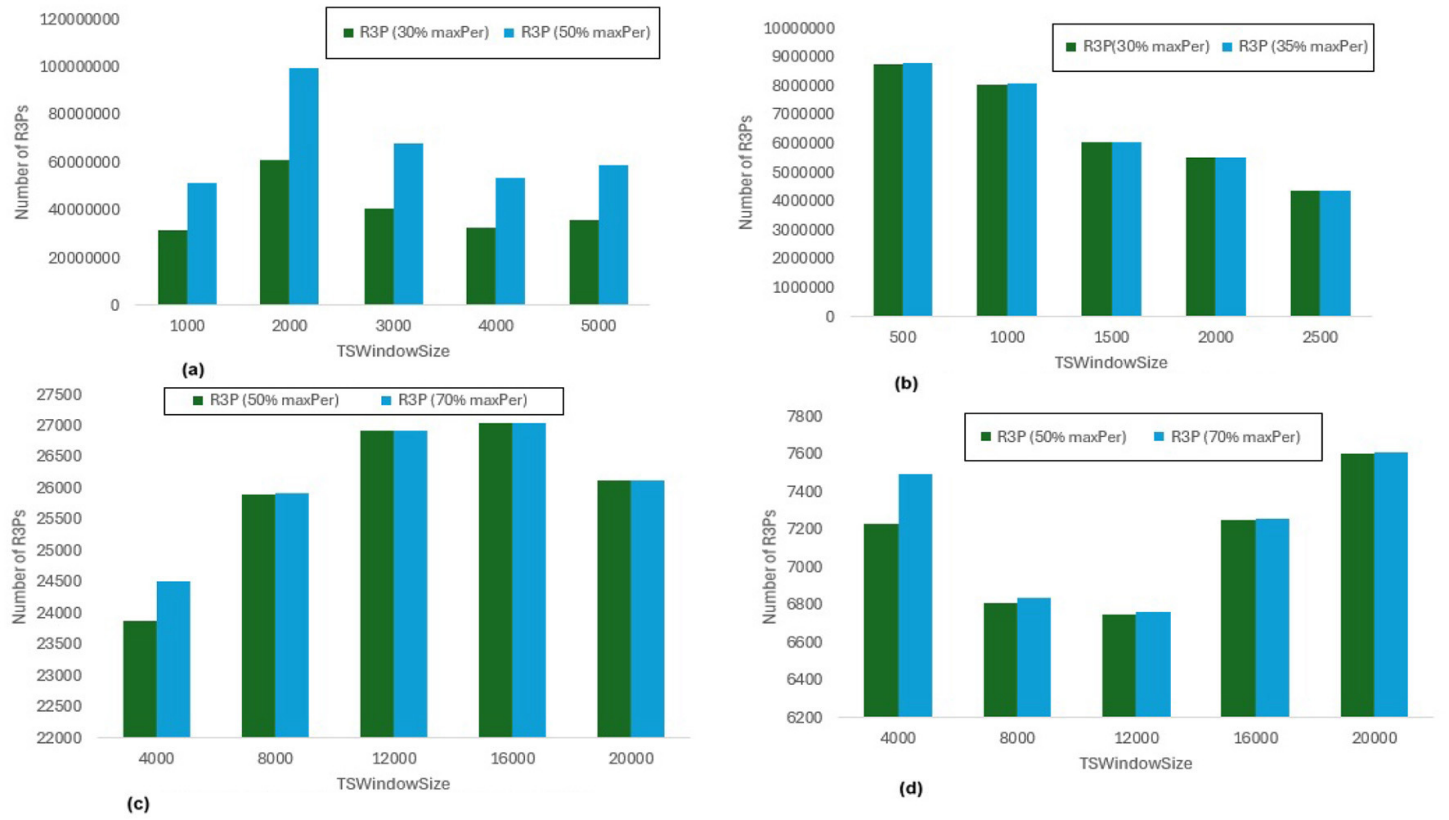
Number of R3Ps generated for *maxPer* variations. **(a)** Accidents, minRarePS = 7%, minFreqPS = 8%. **(b)** Chess, minRarePS = 7%, minFreqPS = 8%. **(c)** T10I4D100K, minRarePS = 0.1%, minFreqPS = 0.5%. **(d)** Retail, minRarePS = 0.1%, minFreqPS = 0.5%.

In Figure 8, *maxPer* threshold, *minFreqPS, TSBatchSize* and *mineBSize* values are kept constant while the runtime performance is observed for different *minRarePS* values. Whereas, Figure 9 represents the corresponding R3Ps generated for this execution setup. On the other side, Figure 10 represent variations of *minFreqPS* values while the *maxPer* threshold, *minRarePS, TSBatchSize* and *mineBSize* values are kept constant. Figure 11 represents the corresponding R3Ps generated for this execution setup. On the contrary, Figure 12 depict execution time performance for different *maxPer* threshold values by keeping *minFreqPS* threshold, *minRarePS* threshold, *TSBatchSize* and *mineBSize* values constant. Whereas, Figure 13 represents the corresponding R3Ps mined for this execution setup. Here, the X-axis represents different *TSWindowSize* values. Whereas the Y-axis shows the runtime in milliseconds (Msec)/Seconds (Sec) in these figures. For the *Accidents* dataset, *TSWindowSize* is varied between 1K to 5K with a 1K difference while the *TSBatchSize* and *mineBSize* values are kept constant as 100 and 600 respectively. While for the *Chess* dataset, the *TSBatchSize* and *mineBSize* values are taken as 10 and 50 respectively and *TSWindowSize* is varied between 0.5K to 2.5K with a difference of 0.5K. In the case of sparse datasets *T10I4D100K* and *Retail*, the *TSBatchSize* and *mineBSize* values are taken as 100 and 600 respectively and *TSWindowSize* is varied between 4K to 20K with a difference of 4K.

#### 6.3.1 Runtime performance when *minFreqPS* and *maxPer* kept constant while varying *minRarePS*

The execution time performance of *Accidents, Chess, T10I4D100K* and *Retail* datasets are shown in Figures 8a–d respectively. The performance improvement is noted by keeping *maxPer* threshold and *minFreqPS* threshold constant and varying *minRarePS* for two different thresholds for each dataset as presented in Table 3. It is observed that, R3P-StreamSWBitVectorMiner outperforms R3PStreamSW-Growth in all the cases as shown in Table 3. Figures 9a–d presents the resultant *R3Ps* produced observing these execution setups. Figure 9b shows that despite *Chess* being a small dense dataset, a vast amount of R3Ps are generated. R3P-StreamSWBitVectorMiner was unable to finish execution because it needed a lot of memory to generate the subsequent R3Ps. As a result, in all the experimentation the number of items in a transaction is lowered to 25 for *Chess* dataset.

**Table 3:** Runtime efficiency comparison of R3P-StreamSWBitVectorMiner for *minRarePS* variations.

**Datasets**	**maxPer (%)**	**minFreqPS (%)**	**minRarePS (%)**	**R3PStreamSW-Growth (%)**
Accidents	30	8	7	93
			7.5	94
Chess	30	8	6	87
			7	91
T10I4D100K	70	0.5	0.1	90
			0.3	90
Retail	70	0.5	0.1	84
			0.3	91

#### 6.3.2 Runtime performance when *minRarePS* and *maxPer* set constant and varying *minFreqPS* threshold value

The execution time performance of *Accidents, T10I4D100K* and *Retail* datasets are shown in Figures 10a–d respectively. The performance improvement is noted by keeping *minRarePS* threshold and *maxPer* threshold constant and varying *minFreqPS* for two different thresholds for each dataset as presented in Table 4. It is noted that, R3P-StreamSWBitVectorMiner outperforms R3PStreamSW-Growth in all the cases as shown in Table 4. Figures 11a–d presents the resultant *R3Ps* produced observing these execution setups.

**Table 4:** Runtime efficiency comparison of R3P-StreamSWBitVectorMiner for *minFreqPS* variations.

**Datasets**	**maxPer (%)**	**minRarePS (%)**	**minFreqPS (%)**	**R3PStreamSW-Growth (%)**
Accidents	30	7	8	93
			9	93
Chess	30	7	8	91
			9	91
T10I4D100K	70	0.1	0.5	90
			10	90
Retail	70	0.1	0.5	84
			10	83

#### 6.3.3 Runtime performance when *minFreqPS* and *minRarePS* kept constant while varying *maxPer* threshold value

The execution time performance of *Accidents, T10I4D100K* and *Retail* datasets are shown in Figures 12a–d respectively. The performance improvement is noted by keeping *minRarePS* threshold and *minFreqPS* threshold constant and varying *maxPer* for two different thresholds for each dataset as presented in Table 5. It is observed that, R3P-StreamSWBitVectorMiner outperforms R3PStreamSW-Growth in all the cases as shown in Table 5. Figures 13a–d presents the resultant R3Ps produced observing these execution setups.

**Table 5:** Runtime efficiency comparison of R3P-StreamSWBitVectorMiner for *maxPer* variations.

**Datasets**	**minRarePS (%)**	**minFreqPS (%)**	**maxPer (%)**	**R3PStreamSW-Growth (%)**
Accidents	7	8	30	93
			50	93
Chess	7	8	30	91
			35	91
T10I4D100K	0.1	0.5	50	90
			70	90
Retail	0.1	0.5	50	83
			70	84

**Influence of** ***minRarePS***, ***minFreqPS*** **and** ***maxPer*** **threshold values:** The following key points are observed from the experiments: (i) In Figure 9, as shown, it is evident that the *minRarePS* variation has a negative effect on the number of generated R3Ps. In particular, at a low threshold, the decrease in *minRarePS*, accelerates the conversion of noisy itemsets to rare 1-itemsets. As the rare 1-itemsets rise, so does the number of R3Ps generated for low *minRarePS* threshold values. Moreover, the execution time increases as the number of R3Ps increases as illustrated in Figure 8. (ii) Conversely, as Figure 11 illustrates, the number of *R3Ps* rises as *minFreqPS* threshold value rises. The variation of *minFreqPS* has shown a favorable outcome. As a result, when the *minFreqPS* threshold value is increased, the time taken also increases very slightly. This is because periodic-1 itemsets are not eliminated immediately even when they exceed the *minFreqPS* threshold value because there is a possibility that their supersets will become PRPs. (iii) As seen in Figure 13, similar observations are made when *maxPer* threshold inceases. An increase in *maxPer* threshold causes aperiodic 1-itemsets to become periodic 1-itemsets, resulting in the generation of more number of *R3Ps*. As seen in Figure 12, there is a slight increase in execution time required with this increase in the number of *R3Ps*. (iv) It is also noted that, in comparison to both *maxPer* and *minFreqPS*, the *minRarePS's* alteration has a greater impact on the execution time.

**Influence of window size** ***TSWindowSize*** **variations:** As seen in Figures 9, 11, 13, the number of one length periodic itemsets that are present during a certain window determines how many R3Ps are generated in the corresponding window. Consequently, when one-length periodic itemsets increase, so does the number of R3Ps in the current window, and vice versa.

### 6.4 Memory consumption

**Figure 14:**
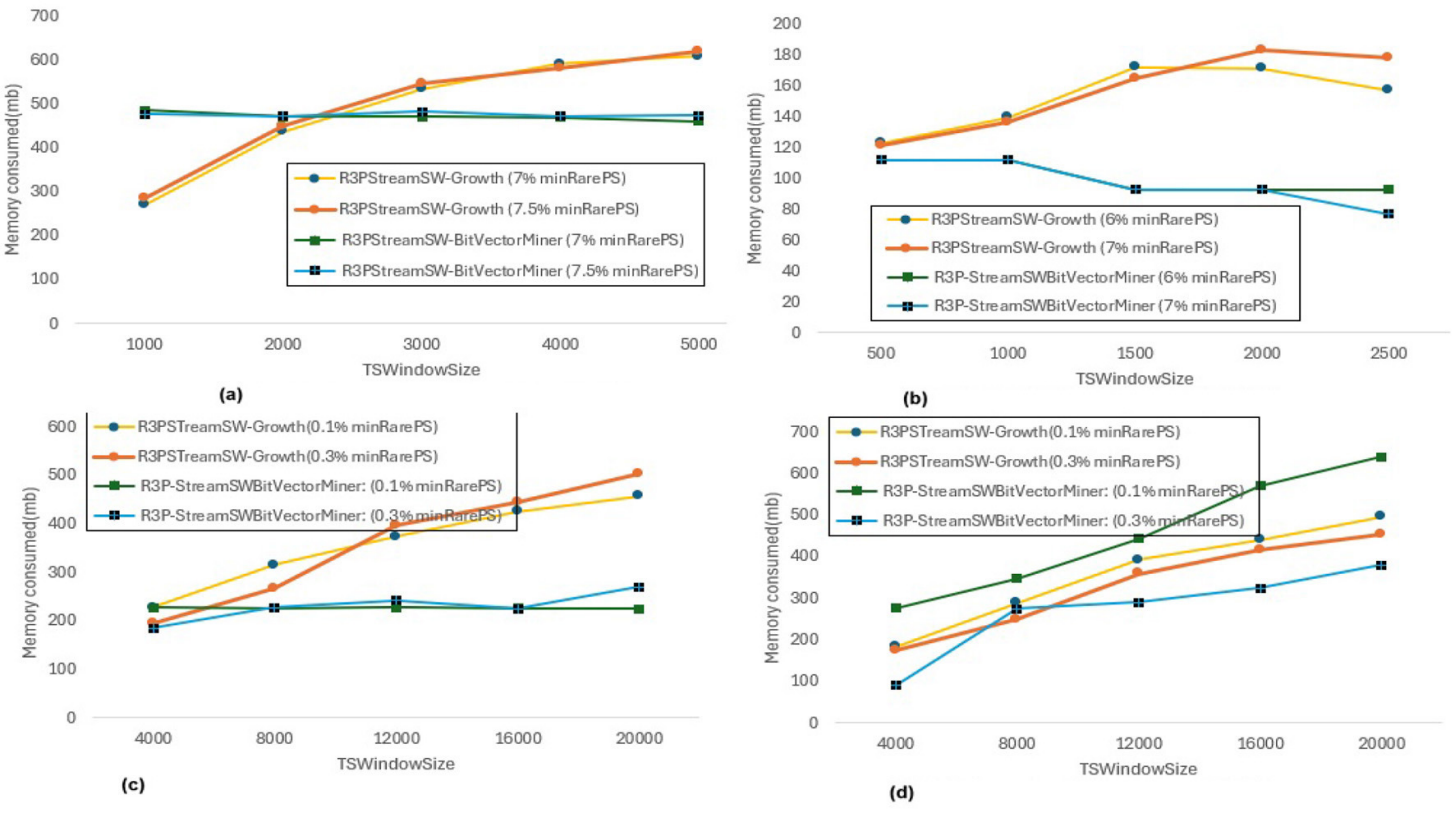
Memory utilization by R3PStreamSW-Growth and *R3P-SreamSWBitVectorMiner* for *minRarePS* variations. **(a)** Accidents, maxPer = 30%, minRarePS = 8%. **(b)** Chess, maxPer = 30%, minRarePS = 8%. **(c)** T10I4D100K, maxPer = 70%, minRarePS = 0.5%. **(d)** Retail, maxPer = 70%, minRarePS = 0.5%.

Section 6.3.1 represents a runtime comparison of proposed rare partial periodic mining frameworks noted by keeping *maxPer* threshold and *minFreqPS* threshold constant and varying *minRarePS* for two different thresholds for each dataset. For the same execution setup, the memory consumed by both proposed frameworks is observed, and it is presented in Figure 14. It is observed that, when R3PStreamSW-Growth is considered, memory utilization rises as the window size increases. It is also observed that R3P-StreamSWBitVectorMiner consumes lesser space in case of *Accidents, Chess and T10I4D100K* by 3%, 4%, 30%, 40%, 30% and 30% for two different variations of *minRarePS* thresholds compared to R3PStreamSW-Growth respectively. The reason behind this is that the transformation of aperiodic-1 items into periodic-1 items or non-rare-1 items into rare-1 itemsets increases memory requirements. Furthermore, as R3PStreamSW-Growth is pattern-growth based, the memory demand increases as the number of conditional pattern-bases and conditional pattern-tree rises. Whereas, it can be observed from Figure 14 that, R3P-StreamSWBitVectorMiner memory requirement is almost constant even when the window size is increased. The reason behind this is, the bit-vector representation consumes almost the same amount of memory irrespective of fluctuation in the count of R3Ps. Compared to R3PStreamSW-Growth, R3P-StreamSWBitVectorMiner consumes lesser memory in all the cases except *Retail* dataset for low *minRarePS*. Even though there is not much difference in the number of temporal transactions of *Retail* and *T10I4D100K*, the number of R3Ps produced is much less in the case of *Retail* compared to *T10I4D100K* for same thresholds considered as shown in Figures 9c, d. This indicates in the case of *Retail*, there are huge aperiodic and noise itemsets. As *Retail* is a large dataset showing high sparse nature, the bit-vector representation may consume more space to represent each item in the case of R3P-StreamSWBitVectorMiner compared to R3PStreamSW-Growth, where the pattern-growth approach helps to remove the noise itemsets faster.

### 6.5 Theoritical znalysis

This section gives the time complexity of R3P-StreamSWBitVectorMiner and R3PStreamSW-Growth algorithms.

**Time complexity analysis of R3PStreamSW-Growth:** Three components are built in this model: R3PStreamSW-Queue, R3PStreamSW-List, and R3PStreamSW-Tree. The following are the different operations carried out by the R3PStreamSW-Growth algorithm:

(i) By accepting the *TSWindowSize* transactions from the stream data, the R3PStreamSW-Tree is built in a single scan during the initial window construction phase. Consider ψ items to be interesting (periodic) and assume they appear in all *TSWindowSize* stream transactions. The construction of R3PStreamSW-List, 3PStreamSW-Tree and R3PStreamSW-Queue are done simultaneously in a single scan by sorting the stream transactions in a pre-defined order. Adding an item at the rear end of the queue has a time complexity of O (1). The time complexity of initial window tree construction is in O (2 (ψ × TSWindowSize)). (ii) Further, in the window sliding phase, initially, *TSBatchSize* number of oldest transactions in the window are removed and new set of *TSBatchSize* number of transactions are added into the R3PStreamSW-Tree. The insertion and deletion has a time complexity of O (2 (ψ × TSBatchSize)). Deleting an item at the front end of the queue has a time complexity of O (1). In addition, at the end of the window sliding phase the R3PStreamSW-List is updated which has a time complexity of O (ψ × TSBatchSize). (iii) To extract R3Ps, the prefix tree is recursively mined in a *dfs* fashion during the mining phase of R3PStreamSW-Growth. The collection of possible itemsets generated is R = 2^ψ^− 1. Finally, to generate the conditional pattern base, R3PStreamSW-List and prefix-tree of α for every considered itemset α that extends an itemset β, R3PStreamSW-Growth traverses the node-links of the R3PStreamSW-List of β. As these structures of β are only visited once, this construction is completed in linear time. The mining has a time complexity of O (ψ × TSBatchSize × R). Hence the *R3PStreamSW-Growth*'s overall time complexity is O (ψ × TSWindowSize × R).

**Time complexity analysis of R3P-StreamSWBitVectorMiner**

(i) After scanning the stream data, all of the one-length items are first saved in bit-vector form in the R3PStreamSWTSList. The construction of a R3PStreamSWTSList has a worst-case time complexity of O (ψ × TSWindowSize). (ii) Moreover, during the window sliding phase, the *TSBatchSize* oldest transactions in the window are first eliminated, and a new set of *TSBatchSize* transactions are added to the R3PStreamSWTSList. To remove the oldest transactions, R3P-StreamSWBitVectorMiner carries out the bitwise left shift operation *TSBatchSize* times, which is an O (1) operation. The time complexity associated with inserting a new batch of *TSBatchSize* number of transactions with support count updates is O (ψ × TSBatchSize). (iii) In order to produce the larger length itemsets, the mining operation applies the logical AND operation on the two current itemsets. Each item is represented by *TSWindowSize* number of bits. The logical AND operation has an O (1) time complexity, regardless of the number of bits involved. Furthermore, the period support calculation considers every bit of each item that requires a maximum *TSWindowSize* number of operations. This algorithm uses the *DFS* method on the itemset lattice. The collection of possible itemsets generated is R = 2^ψ^− 1. Consequently, the time required to generate all possible interesting itemsets is O (R × TSWindowSize). Therefore, the total time complexity of R3P-StreamSWBitVectorMiner is O (R × TSWindowSize).

## 7 Conclusion

In this paper, novel tree-based framework *R3PStreamSW-Growth* and list-based framework *R3P-StreamSWBitVectorMiner*, innovative sliding window-based techniques are introduced to capture rare partial periodic patterns from temporal stream data. Two distinct support thresholds *minRarePS* and *minFreqPS* are employed in addition to *maxPer* threshold measure to regulate the number of cyclic repetitions and eliminate noisy patterns. In order to maintain the sliding window, the user accepts window size - *TSWindowSize* and batch size - *TSBatchSize* values. In addition, *mineBSize*, a user-specified value, decides the time at which mining is performed after how many batches of the sliding window process. *R3PStreamSW-Growth* maintains a *R3PStreamSW-List* structure in which the current timestamp window's partial periodic one-length patterns are preserved. This helps to reduce the ample search space by removing one-length aperiodic patterns. Furthermore, to capture all time-stamped data from the current window stream, a single scan *R3PStreamSW-Tree* is built. In addition, a queue structure called *R3PStreamSW-Queue* points to the nodes of *R3PStreamSW-Tree* with timestamp data, thus speeding up traversal during window sliding phase. As new stream data are added and older transactions are eliminated when window slides, *R3PStreamSW-Tree* is always in a ready-to-mine condition. During the mining phase, *R3PStreamSW-Growth* employs a divide-and-conquer strategy, which generates a massive number of conditional pattern trees which effects mining performance. To overcome this, a list-based framework, *R3P-StreamSWBitVectorMiner* is proposed to extract rare partial periodic patterns from the temporal stream data. The current window stream data are transformed into bit-vector and stored in an efficient data structure named *R3PStreamSWTSList* which helps in pruning non-periodic itemsets. The findings showed that when a dense dataset *Accidents* is considered for *minRarePS, minFreqPS* and *maxPer* threshold variations, *R3P-StreamSWBitVectorMiner* outperformed *R3PStreamSW-Growth* by about 93%. Similarly, when the sparse dataset *T10I4D100K* is taken into account, *R3P-StreamSWBitVectorMiner* exhibits a 90% boost in performance. This demonstrated that on a range of synthetic, real-world, sparse, and dense datasets for different thresholds, *R3P-StreamSWBitVectorMiner* is significantly faster than *R3PStreamSW-Growth*. In addition, it is also observed that *R3P-StreamSWBitVectorMiner* is memory-efficient compared to *R3PStreamSW-Growth* in most of the cases. In contrast, it is observed that for a highly sparse large dataset, where the number of aperiodic and noise items are more, *R3P-StreamSWGrowth* consumes lesser memory. As *R3P-StreamSWBitVectorMiner* represents the transaction ids in bit-vector form, compressing the tidset may increase space efficiency.

The proposed frameworks are restricted to extract rare partial periodic patterns from temporal stream data based on the *maxPer* threshold value. To overcome this limitation, as a future work alternative periodic support metrics can be applied as per the user requirements. In addition, the proposed frameworks may be employed to extract rare periodic patterns by considering suitable real-world applications such as e-business, cybersecurity, healthcare, and network traffic data. Similarly, the proposed models could be integrated with existing machine learning prediction models to extract significant information. The proposed methods mine rare partial periodic patterns considering the temporal stream data. However, an in-depth study may be carried out to find the significant associations that exist among the rare partial periodic patterns generated. Further, the proposed frameworks can be enhanced to mine high-utility itemsets from stream data. Additionally, the proposed methods can be extended to consider multivariate time series datasets.

## Data Availability

Publicly available datasets were analyzed in this study. This data can be found here: https://github.com/udayRage/pykit_old/tree/master/Datasets.
